# LncRNA-*Safe* contributes to cardiac fibrosis through *Safe*-*Sfrp2*-HuR complex in mouse myocardial infarction

**DOI:** 10.7150/thno.33920

**Published:** 2019-09-25

**Authors:** Kaili Hao, Wei Lei, Hongchun Wu, Jie Wu, Zhuangzhuang Yang, Shiping Yan, Xing-Ai Lu, Jingjing Li, Xue Xia, Xinglong Han, Wenbo Deng, Guisheng Zhong, Zhen-Ao Zhao, Shijun Hu

**Affiliations:** 1 Department of Cardiovascular Surgery of the First Affiliated Hospital & Institute for Cardiovascular Science, State Key Laboratory of Radiation Medicine and Protection, Medical College, Soochow University, Suzhou 215000, China; 2 Fujian Provincial Key Laboratory of Reproductive Health Research, School of Medicine, Xiamen University, Xiamen 361102, China; 3 iHuman Institute, School of Life Science and Technology, ShanghaiTech University, Shanghai 210021, China

**Keywords:** non-coding RNA, fibrosis, cardiac remodeling

## Abstract

**Rationale**: As a hallmark of various heart diseases, cardiac fibrosis ultimately leads to end-stage heart failure. Anti-fibrosis is a potential therapeutic strategy for heart failure. Long noncoding RNAs (lncRNAs) have emerged as critical regulators of heart diseases that promise to serve as therapeutic targets. However, few lncRNAs have been directly implicated in cardiac fibrosis.

**Methods**: The lncRNA expression profiles were assessed by microarray in cardiac fibrotic and remote ventricular tissues in mice with myocardial infarction. The mechanisms and functional significance of lncRNA-*AK137033* in cardiac fibrosis were further investigated with both *in vitro* and *in vivo* models.

**Results**: We identified 389 differentially expressed lncRNAs in cardiac fibrotic and remote ventricular tissues in mice with myocardial infarction. Among them, a lncRNA (*AK137033*) we named *Safe* was enriched in the nuclei of fibroblasts, and elevated in both myocardial infarction and TGF-β-induced cardiac fibrosis. Knockdown of *Safe* prevented TGF-β-induced fibroblast-myofibroblast transition, aberrant cell proliferation and secretion of extracellular matrix proteins *in vitro*, and mended the impaired cardiac function in mice suffering myocardial infarction. *In vitro* studies indicated that knockdown of *Safe* significantly inhibited the expression of its neighboring gene *Sfrp2*, and vice versa. The *Sfrp2* overexpression obviously disturbed the regulatory effects of *Safe* shRNAs in both the *in vitro* cultured cardiac fibroblasts and myocardial infarction-induced fibrosis. Dual-Luciferase assay demonstrated that *Safe* and *Sfrp2* mRNA stabilized each other via their complementary binding at the 3'-end. RNA electrophoretic mobility shift assay and RNA immunoprecipitation assay indicated that RNA binding protein HuR could bind to *Safe*-*Sfrp2* RNA duplex, whereas the knockdown of *HuR* dramatically reduced the stabilization of *Safe* and *Sfrp2* mRNAs, down-regulated their expression in cardiac fibroblasts, and thus inhibited TGF-β-induced fibrosis. The *Safe* overexpression partially restrained the phenotype change of cardiac fibroblasts induced by *Sfrp2* shRNAs, but not that induced by *HuR* shRNAs.

**Conclusions**: Our study identifies *Safe* as a critical regulator of cardiac fibrosis, and demonstrates *Safe*-*Sfrp2*-HuR complex-mediated* Sfrp2* mRNA stability is the underlying mechanism of *Safe*-regulated cardiac fibrosis. Fibroblast-enriched *Safe* could represent a novel target for anti-fibrotic therapy in heart diseases.

## Introduction

Injury in any organ triggers a complex cascade of biological responses, culminating in tissue fibrosis 1. As a hallmark of various heart diseases, cardiac fibrosis ultimately leads to heart failure, the predominant cause of morbidity and mortality in the world. Cardiac fibroblasts are now recognized as a main contributor of cardiac fibrosis 2. In response to heart injury, cardiac fibroblasts are activated to proliferate, differentiate into myofibroblasts, and secret extracellular matrix (ECM) proteins such as type I collagen 3. Despite these adaptive features in the initial stage, prolonged injurious stimuli may cause cellular dysfunction, scar formation, and ultimately organ failure 1. Anti-fibrosis has emerged as a potential therapeutic strategy for heart failure caused by cardiac fibrogenesis. However, due to our limited understanding of the mechanisms underlying disease-induced fibrosis, no effective therapies have been developed to target cardiac fibrogenesis seen in clinics 4.

Multiple cytokines including transforming growth factor-β (TGF-β) have emerged as pivotal profibrotic factors. In response to tissue injury, TGF-β activates fibroblast proliferation and differentiation into myofibroblast through both canonical and non-canonical TGF-β signaling 5, 6. Secreted frizzled-related protein 2 (*Sfrp2*) was recently reported to play a critical role on cardiac fibrosis after myocardial infarction (MI), partially through activating bone morphogenic protein 1 (BMP1) to initiate a feed-forward loop between TGF-β and BMP1 7-9. However, the signals stimulating SFRP2 production are still to be defined.

Emerging evidence indicates the dynamic expression of long noncoding RNAs (lncRNAs) in the developmental and diseased heart, suggesting a profound biological function 10, 11. However, the functions of lncRNAs in cardiac fibrosis are still not clearly characterized 12. To date, only few lncRNAs, including *Wisper*, *Meg3*, and *MIAT*, have been implicated in cardiac fibrosis 13-15. Therefore, further investigations of lncRNA functions in heart fibrosis and the underlying mechanisms are needed to identify better targets to treat cardiac remodeling.

Here, we find 389 lncRNAs that are differentially expressed in cardiac fibrotic tissue and remote myocardial tissue after myocardial infarction, and we further identify *AK137033*, a lncRNA named *Safe* in our study, as a critical regulator of MI-induced cardiac fibrosis. Our studies indicate that nucleus-enriched *Safe* could increase the mRNA stability of *Sfrp2* through the formation of *Safe*-*Sfrp2*-HuR complex, and promote its protein expression in cardiac fibroblast. Knockdown of *Safe* mitigates both TGF-β and MI-induced cardiac fibrosis, and improves heart function by inhibiting *Sfrp2*-mediated activation of fibroblast proliferation, fibroblast-myofibroblast transition, and deposition of ECM.

## Methods

***Animal studies***. All experimental protocols involving animals in this study were approved by the Laboratory Animal Research Committee of Soochow University. Adult CD1 mice (8-10 weeks old) were purchased from Shanghai Laboratory Animal Center at the Chinese Academy of Sciences (Shanghai, China). Myocardial infarction was induced in adult female CD1 mice by ligation of the mid-left anterior descending artery (LAD), and confirmed by myocardial blanching and electrocardiographic changing. Due to the different maladaptive remodeling and survival rate of males and females post MI, we used only female mice in this study to exclude the gender influence on cardiac function. Mice were randomly assigned to different groups. Lentiviral particles (1×10^9^ TU/mL, 10 μL) carrying scramble control shRNA, or *Safe*-specific shRNA, were injected, in combination with/without lentivirus overexpressing *Sfrp2*, at 3 injection sites in the infarcted area immediately post MI induction. Echocardiography was performed before and after LAD ligation at indicated time points, using the Visual Sonics Vevo2100 system (Toronto, ON, Canada) equipped with a medium frequency (30 MHz) MS-400 transducer. Ultimately, heart tissues were harvested by either pre-chilled in liquid nitrogen or embedded in Optimal Cutting Temperature compound (Sakura Finetechnical, Tokyo, Japan), and stored at -80 °C for further studies. All the parameter measurements and analyses were blinded.

***Microarray analysis***. Following RNA extraction, total RNAs were linearly amplified and labeled with Cy3 using the Quick Amp labeling kit (Agilent Technologies, Santa Clara, CA, USA). The labeled cDNAs were hybridized to lncRNA expression microarray slide, and then scanned using the Agilent DNA Microarray Scanner using the recommended settings. The acquired array images were analyzed with Agilent Feature Extraction Software (version 10.7.3.1), followed by quantile normalization and background correction using the Agilent GeneSpring GX software (version 11.5.1). Significant differential expression was identified using criteria based on arbitrary fold change (≥ 2) and p-value (p < 0.05).

***Cell culture***. Cardiac fibroblasts were isolated from adult mouse hearts. Briefly, cardiac ventricles from adult CD1 mice were digested with 0.1% collagenase II (Sigma, St. Louis, MO, USA) three times at 15-minute intervals. Cell suspensions from each interval digestion were filtered through a 70-µm cell strainer, and washed for three times. Cell pellets were resuspended in DMEM/F12 medium supplemented with 1% FBS, and pre-plated for 2 hours on 60-mm dishes in a humidified incubator supplied with 5% CO_2_. The attached cells, which were cardiac fibroblasts, were then washed with PBS, and ultimately cultured in DMEM/F12 medium supplemented with 10% FBS, 100 U/mL penicillin, and 100 µg/mL streptomycin.

***Myofibroblast transformation*.** When cell density reached 60%-70%, cardiac fibroblasts were pretreated with serum-free DMEM/F12 medium for 2 hours, and then induced with 20 ng/mL recombinant TGF-β (PeproTech, Rocky Hill, NJ, USA) for 72 hours. Successful induction of myofibroblasts was verified by the abundant expression of myofibroblastic markers α-SMA and COL1A1. For functional studies, cardiac fibroblasts were infected with lentiviral particles carrying *Safe*, *Sfrp2*, *HuR* or scrambled shRNA sequences, followed by TGF-β-mediated induction of myofibroblast.

***Rapid amplification of cDNA ends (RACE).*** Total RNAs were extracted from cardiac fibroblasts, and were polyadenylated with Poly(A) Polymerase (TaKaRa, Kusatsu, Japan). First strand cDNAs were synthesized from the poly(A)-tailed RNAs using the SMARTer® RACE 5'/3' kit (TaKaRa) as per the manufacturer's instructions. The 5'-RACE and 3'-RACE PCRs were performed using the gene special primers (GSP, Table S1) and universal primer mix (SMARTer® RACE 5'/3' kit), respectively. The RACE products were cloned into the T vector (TaKaRa), followed by Sanger sequencing.

***Construction of shRNA, CRISPR/Cas9 and overexpression lentiviral vectors*.** To construct lentiviral vectors targeting *Safe, Sfrp2* or *HuR*, shRNA sequences listed in Table S2 were synthesized and cloned into the unique BamHI/EcoRI sites in pGreenPuro™ Vector (System Biosciences, Palo Alto, CA, USA), and the scramble sequence was applied for shRNA knockdown control. Lentiviral CRISPR/Cas9 vectors targeting *Safe* gene were constructed by subcloning *Safe*-targeting gRNAs (Table S2) into the lentiCRISPRv2 vector (Addgene, Watertown, MA, USA), and the lentiCRISPRv2 vector without insertion was used as CRISPR knockdown control for subsequent studies^16^. To construct the lentiviral overexpression vectors, the full-length *Safe* nucleotide fragments or *Sfrp2*-coding regions were amplified using primers listed in Table S2, and inserted into the EcoRI/BamHI sites in the pCDH-CMV-MCS-EF1-copGFP vector (SBI, San Francisco, CA, USA), respectively. The empty vector was used as overexpression control. These lentiviral vectors were then transfected into 293T cells, along with the lentiviral packaging plasmid (psPAX2) and VSV-G envelope expressing plasmid (pMD2.G). Lentiviral particles released into the cell culture medium were collected and concentrated with PEG 8000.

***Cell proliferation assay*.** Cell proliferation was assessed with Cell Counting Kit-8 (Dojindo, Kumamoto, Japan) as per the manufacturer's instructions. In brief, cardiac fibroblasts (1×10^4^ cells/well) were plated onto 96-well plates, followed by TGF-β treatment for 72 hours. At indicated time points, cells in each well were incubated in a culture medium containing 10 μL CCK-8 solution for 4 hours. The absorbance at 450 nm was then measured using Synergy H1 Hybrid Multi-Mode Microplate Reader (BioTek, Winooski, VT, USA).

***Collagen contraction assay*.** Fibroblast contractile activity was assessed by collagen contraction assay as per previous description 17. In brief, 4×10^4^ cells (400 μL) were mixed with 200 μL of rat tail type I collagen matrix (3 mg/mL, R&D systems, Minneapolis, MN, USA), and poured into a well of 24-well plates. After solidification at 37 °C for 30 minutes, cells were cultured in DMEM/F12 medium supplemented with 1% FBS. The diameter changes of collagen gels were recorded after a 24-hour culture.

***Evaluation of fibrosis area.*** Mouse hearts were cut into 10 μm-thick cross sections along the center of the MI zone, and then stained by Masson's trichrome staining (Solarbio, Beijing, China). The percentage of fibrotic tissue area (blue area) in the left ventricular wall was calculated with ImageJ software 18.

***BMP1 enzyme activity assay*.** BMP1 enzyme activity was measured using a fluorescent assay as described by the manufacturer's instruction (R&D Systems). Following different treatments, cardiac fibroblasts were incubated with collection buffer (25 mM HEPES, 0.1% Brij-35, pH 7.5) for 6 hours. The supernatant samples were then diluted to the same concentration with collection buffer. The diluted supernatant samples (50 μL each reaction) were mixed with an equal volume of fluorogenic substrate (Mca-Tyr-Vla-Asp-Ala-Pro-Lys(Dnp)-OH, R&D systems), and subsequently measured using a fluorescent plate reader (BioTek) at 320 nm in excitation and 405 nm in emission, respectively. The standard curve was derived using the free fluorescent product (Mca-Pro-Leu-OH, Bachem, Essex, UK), and used to calibrate the readings.

***In vitro transcription/translation assay***.* In vitro* translation of *Safe* was conducted using TnT® Quick Coupled Transcription/Translation Kit (Promega, Madison, WI, USA). The full-length *Safe* fragments were amplified from the fibroblast cDNAs with specific primers containing the T7 RNA polymerase promoter (Table S1). These PCR-generated fragments were then added to an aliquot of the TnT® Quick Master Mix and incubated in a 50 μl reaction volume for 60-90 minutes at 30°C. The Luciferase Control DNAs carrying the T7 RNA polymerase promoter were used as a positive control for *in vitro* translations. The translation products were separated by SDS-PAGE and detected with the Transcend^TM^ Non-Radioactive Translation Detection System (Promega).

***Fluorescence in situ hybridization (FISH).*** The RNAscope 2.0 assay (Advanced Cell Diagnostics, Newark, Canada) was performed for *in situ* detection of *Sfrp2* and *Safe* RNAs in cardiac fibroblasts. Briefly, cardiac fibroblasts were fixed with 10% formalin for 30 minutes at room temperature, digested with protease and followed by hybridizations with probes targeting *Sfrp2* and *Safe* RNAs, respectively. Cells were then counterstained with DAPI, and the fluorescence signals were visualized using the ZEISS LSM 880 Confocal Laser Scanning Microscope (ZEISS, Oberkochen, Germany).

***Subcellular fractionation***. Nuclear/cytosol isolation kit was purchased from Biovision (Milpitas, CA, USA). According to the manufacturer's instruction, 1×10^7^ cardiac fibroblasts were resuspended in 0.2 mL Cytosol Extraction Buffer-A Mix. Then 11 µL of ice-cold cytosol extraction Buffer-B was added into the cell suspension, followed by vigorous vortex of samples. After centrifugation, the supernatant was collected as the cytosolic fraction. The pellet was then re-suspended in 100 µL of ice-cold Nuclear Extraction Buffer Mix, and vortexed thoroughly. The extract was then centrifuged, and the supernatant was collected as the nuclear fraction.

***Quantitative real-time PCR analysis (qRT-PCR)*.** Invitrogen^TM^ TRIzol^TM^ reagent (Invitrogen, Carlsbad, CA, USA) was used to extract total RNA from tissues, cells, and subcellular fractions. An aliquot of total RNA (500 ng) was reverse-transcribed into single-strand complementary DNA using the TaKaRa PrimeScript^TM^ RT Reagent Kit (Clontech, Mountain View, CA, USA). RNA expression was detected and analyzed using the StepOnePlus^TM^ Real-Time PCR System (Applied Biosystems, Foster City, CA, USA). Data were normalized to *18S* rRNA, and analyzed using 2^-ΔΔCT^ method. All primer sequences were listed in Table S3.

***RNA electrophoretic mobility shift assay (RNA EMSA)*.** To verify the binding of HuR with *Safe*-*Sfrp2* RNA duplexes, RNA EMSA was performed using LightShift^TM^ Chemiluminescent RNA EMSA Kit (Pierce, Rockford, IL, USA) with nuclear extract of cardiac fibroblasts. Biotin-labeled single-stranded RNA probes or unlabeled complementary RNA fragments were synthesized corresponding to consensus RNA binding sites of HuR. Protein-lncRNA binding reactions were performed in the REMSA binding buffer containing nuclear proteins of fibroblasts along with 10 pM of biotin-labeled single-stranded RNA probe, or the annealed RNA duplexes with biotin-labeling on the same strand. The reactions were then loaded onto 6% polyacrylamide gel and transferred to a positive-charged nylon membrane (Roche, Mannheim, Germany). After UV cross-linking, the biotin-labeled RNA probes were detected using HRP-conjugated streptavidin, and visualized with ECL reagents. For supershift analysis, 200 ng of anti-HuR antibody (Cell Signaling Technology, Danvers, MA, USA) or IgG were added into the Protein-lncRNA binding reactions, followed by gel electrophoresis and ECL visualization. The probe sequences were listed in Table S4.

***RNA immunoprecipitation (RIP)*.** Following UV cross-linking, the nuclei of cardiac fibroblasts were isolated and sonicated in nuclear lysis buffer (50 mM Tris-HCl pH 8.1, 150 mM NaCl, 0.1% NP-40, 1 mM DTT). The nuclear extracts were then incubated with anti-HuR antibody or IgG, along with Magna ChIP Protein G Magnetic Beads (EMD Millipore, Darmstadt, Germany). The protein-RNA complexes on the beads were successively washed with wash buffer I and wash buffer II, and resuspended in TRIzol reagent for RNA extraction. Reverse transcription and PCR were then conducted using TaKaRa PrimeScript^TM^ RT Reagent Kit and SYBR Premix Ex Taq kit (Clontech).

***Western blot*.** Total protein was harvested from cells subjected to different treatments, using RIPA lysis buffer supplemented with protease inhibitors cocktail. The protein samples were then subjected to SDS-polyacrylamide gel electrophoresis (SDS-PAGE) and transferred to PVDF membranes (EMD Millipore). The membranes were probed with antibodies of interest and visualized by Phototope-HRP Western Blot Detection kit (Cell Signaling Technology).

***Luciferase reporter assay*.** The respective fragments of *Safe* and the 3'-UTR of *Sfrp2* were amplified using primers listed in Table S1, and inserted into the pGL3-Control vector downstream of the Firefly luciferase reporter gene. NIH 3T3 cells were co-transfected with recombinant pGL3-Control vectors containing various fragments, along with pRL-TK plasmid containing Renilla luciferase. After a 24-hour culture, Firefly and Renilla luciferase activities in each cell lysate sample were measured using the Dual-Luciferase Reporter Assay System (Promega). The relative Firefly luciferase activities were normalized to the respective Renilla luciferase activities in each sample.

***Immunofluorescence*.** After fixation with 4% paraformaldehyde in PBS, cells were blocked with 5% donkey serum in PBS containing 0.1% Triton X-100 for 1 hour at room temperature, and incubated with primary antibodies at 4ºC overnight. The cells were further incubated with fluorescent-labeled secondary antibodies for 1 hour at room temperature, counterstained with DAPI (5 μg/mL), and then observed under a fluorescence microscope. The primary antibodies used in this study include α-SMA (Sigma), pH3 (Santa Cruz, USA), and HuR (Proteintech, Wuhan, China).

***Statistical analysis*.** Comparisons between two groups were analyzed using Student's *t*-test. Comparisons in multiple groups were analyzed with one-way analysis of variance (ANOVA) or two-way repeated-measures analysis of variance with the Bonferroni post hoc test. Statistical significance was denoted by a *p* value of less than 0.05. All data were presented as mean ± SEM. All experimental assays were performed at least three times.

## Results

### 1. LncRNA-*Safe* is a fibroblast-enriched lncRNA and associated with cardiac fibrosis

**Figure 1:**
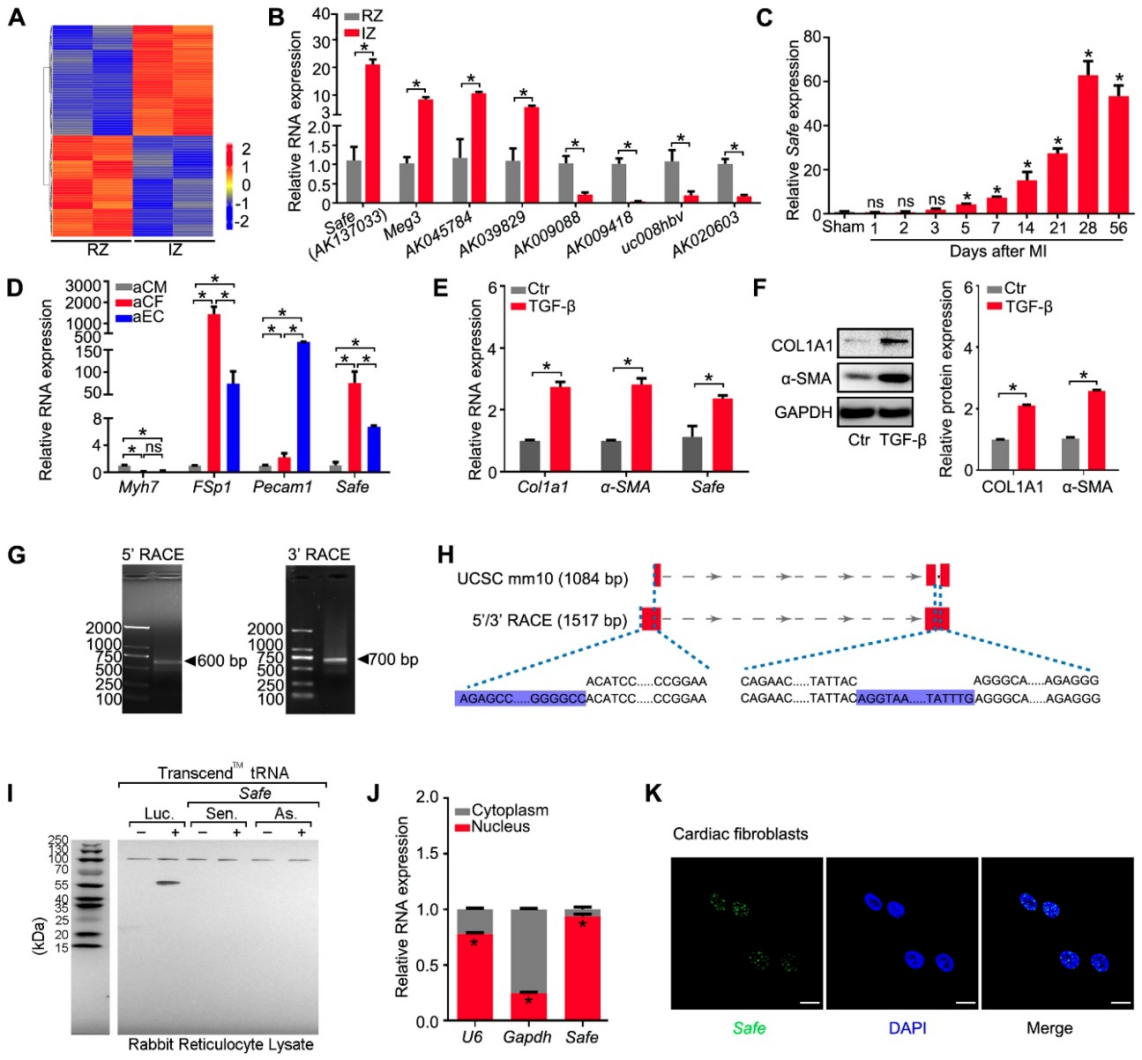
***Safe* is a fibroblast-enriched lncRNA and associated with cardiac fibrosis.** (A) Heatmap showing 389 lncRNAs differentially expressed in tissues from the infarction zone (IZ) and non-infarcted remote zone (RZ) at day 14 post MI (fold ≥ 2, *p*<0.05). (B) qRT-PCR validation of four upregulated lncRNAs and four downregulated lncRNAs in cardiac samples from IZ and RZ of infarcted hearts (n=3). (C) qRT-PCR analysis of *Safe* expression in healthy (sham) or infarcted myocardium at indicated days post MI (n=3). (D) qRT-PCR detection of *Safe* expression in adult cardiomyocytes (aCM), adult cardiac fibroblasts (aCF) and adult endothelial cells (aEC). *Myh7* (myosin heavy chain 7), *FSp1* (fibroblast specific protein-1) and *Pecam1* (Platelet endothelial cell adhesion molecule-1) were used as markers of aCM, aCF and aEC, respectively (n=3). (E) qRT-PCR detection of *Safe*, *Col1a1* and *α-SMA* expression in TGF-β-treated cardiac fibroblasts (n=3). (F) Representative western blot analysis and relative densitometric quantification of COL1A1 and α-SMA protein levels in cardiac fibroblasts with TGF-β treatment (n=3). (G) Agarose gel electrophoresis of the 5' and 3' RACE amplification products. (H) Schematic presentation of full-length *Safe* showing the extended regions identified by RACE (blue). The full-length of *Safe* was 1517 base pairs with two exon regions, which was different from the sequence information provided by UCSC mm10 showing 1048 base pairs in length and three exon regions. (I)* In vitro* translation of *Safe* sense or antisense transcript. Luciferase (Luc) was used as a positive control. (J) Subcellular localization of *Safe* in cytoplasm and nucleus of cardiac fibroblasts (n=3). The gene *U6* and *Gapdh* were respectively used as nuclear and cytoplasmic RNA markers. (K) Representative images of RNA FISH showing nuclear localization of *Safe* (green) in cardiac fibroblasts. The nucleuses were counterstained with DAPI (blue). Scale bar indicates 20 μm. Data are presented as mean ± SEM and analyzed using Student's *t*-test or one-way ANOVA; *p < 0.05, and ns, not significant.

To explore the potential lncRNAs involved in cardiac fibrosis, we performed microarray-based transcriptome analyses on mouse infarcted heart 2 weeks post LAD ligation, and identified a total of 389 lncRNAs differentially expressed between tissues from infarct zone (IZ) and remote zone (RZ), as shown in Figure 1A and Table S5. The qRT-PCR results confirmed the specific expression patterns of eight randomly selected lncRNAs, including a known heart-related lncRNA-*Meg3* (Figure 1B). Among them, a lncRNA (*AK137033*) that we named *Sfrp2* antisense as fibrosis enhancer (*Safe*) showed a continually increased expression in the mouse infarcted heart from day 5 to day 56 post MI, the stage of cardiac fibrogenesis and pathological remodeling (Figure 1C). Compared with samples from the border zone (BZ), RZ and normal myocardium, *Safe* transcripts were significantly enriched in the fibrotic tissues from IZ (Figure 1B, Figure S1A-B), which suggests the potential involvement of *Safe* in MI-induced cardiac fibrosis.

Because fibroblast accumulation is a principal determinant of cardiac fibrosis, we quantified the expression of *Safe* in cardiac cells, and confirmed that *Safe* transcripts were greatly enriched in fibroblasts, and showed the lowest expression in cardiomyocytes in both neonatal and adult mouse hearts (Figure 1D, Figure S1C). In addition, the *Safe* transcripts were also abundantly expressed in fibroblasts from other tissues such as lung and skin (Figure S1D). We further assessed the expression level of *Safe* in TGF-β-induced cardiac fibrosis *in vitro*. After a 72-hour treatment of TGF-β, a central mediator of fibrogenesis, the primary adult cardiac fibroblasts displayed an increased expression of myofibroblastic markers such as COL1A1 and α-SMA (Figure 1E-F), which indicates the successful induction of cardiac fibrosis. Most importantly, the expression of *Safe* was also fundamentally induced in fibroblasts treated with TGF-β (Figure 1F).

In order to characterize *Safe* RNAs, we performed rapid amplification of cDNA ends (RACE) experiments and sequenced to amplification products from both 5'-RACE and 3'-RACE (Figure 1G, Table S6). The sequence alignment results indicated that the full-length of *Safe* RNA is 1517 nucleotides (nt) with two exons, rather than 3 exons as identified previously in *AK137033* (Figure 1H). The two exons are 705 nt and 812 nt in length respectively, and separated by an intron of 14909 nt length. We presumed the two-exon transcript represents a novel isoform of *AK137033*. However, only the two-exon transcript was detected in the cardiac fibroblasts (Figure S1E). Although the NCBI ORF Finder predicated a total of 17 open-reading frames (ORFs) (Table S7), we confirmed that the full-length *Safe* gene does not encode for a detectable protein using the *in vitro* transcription/translation assay (Figure 1I). Quantification of nucleus/cytoplasm RNAs revealed that *Safe* transcripts presented predominantly in the nuclear fraction of fibroblasts (Figure 1J, Figure S1D), which was further confirmed by the FISH experiments (Figure 1K).

Collectively, these data indicate that *Safe* is abundantly expressed in the nuclei of fibroblasts and elevated in both MI and TGF-β-induced cardiac fibrosis. We thus speculate that *Safe* might be a potential lncRNA regulator of cardiac fibrosis.

### 2. Suppression of *Safe* prevents TGF-β-induced cardiac fibrosis *in vitro*

**Figure 2:**
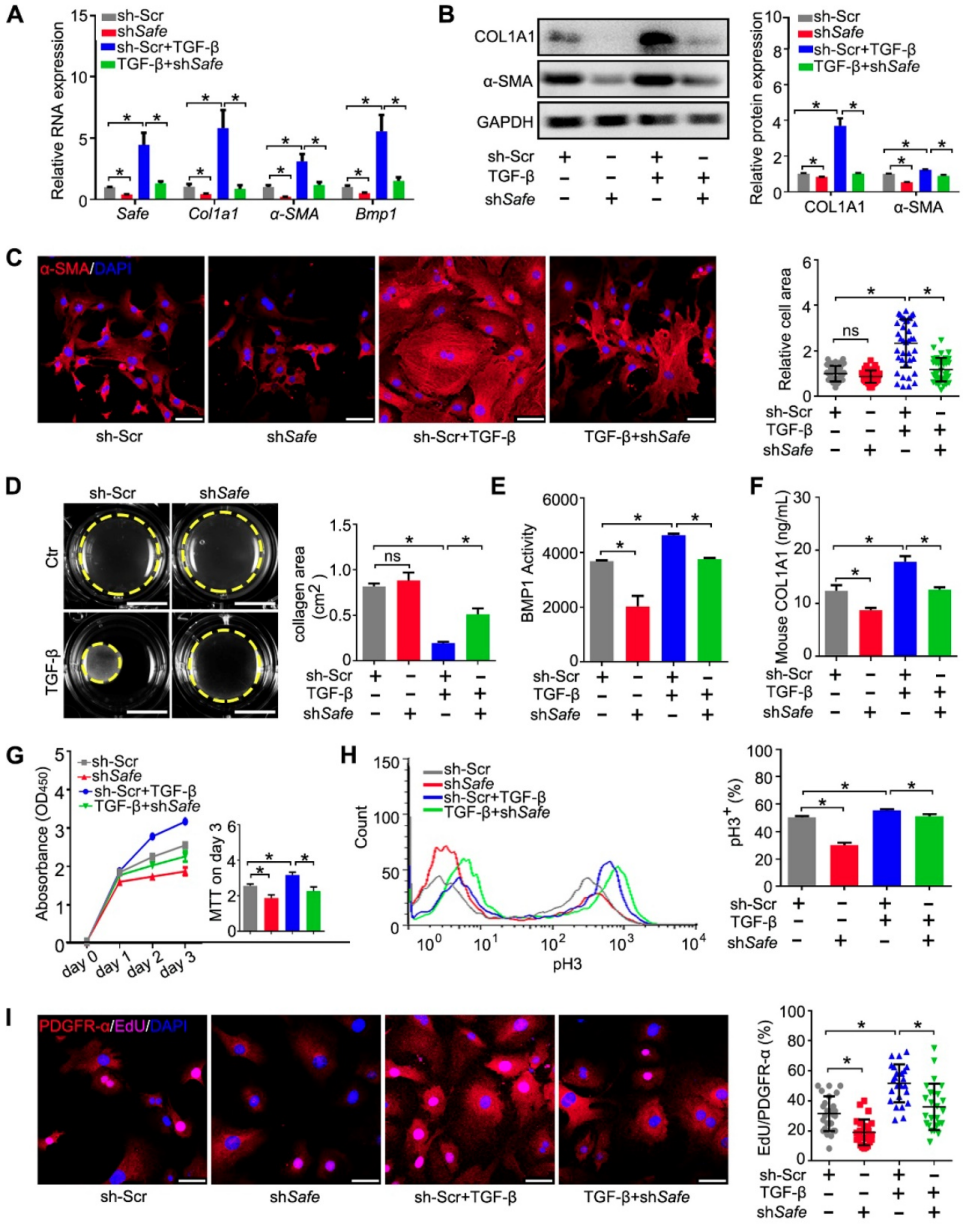
** Suppression of *Saf*e prevents TGF-β-induced cardiac fibrosis *in vitro*.** (A) mRNA expression of *Safe*, *Col1a1*, *α-SMA* and *Bmp1* in TGF-β-untreated or TGF-β-treated cardiac fibroblasts after *Safe* knockdown (n=3). (B) Western blot analysis and relative densitometric quantification of COL1A1 and α-SMA protein levels in TGF-β-untreated or TGF-β-treated cardiac fibroblasts after *Safe* knockdown (n=3). (C) Representative images of immunofluorescence staining for α-SMA (n=5) and quantification of the relative cell areas of cardiac fibroblasts after treatments as indicated (n=40). The nuclei were counterstained with DAPI (blue). Scale bar indicates 50 μm. (D) Representative images of collagen gel contraction for 24 hours and quantification of collagen area inside the dashed circles (n=3). Scale bar indicates 0.5 cm. (E) BMP1 protein enzyme activity in the supernatant of cultured fibroblasts after indicated treatments (n=3). The excitation wavelength is 320 nm, and the emission wavelength is 405 nm. (F) ELISA assay of COL1A1 protein in the supernatant of cultured fibroblasts after indicated treatments (n=3). (G) CCK-8 assay of cardiac fibroblasts with TGF-β-untreated or TGF-β-treated showing repressed cell proliferation by *Safe* knockdown. The cell proliferation rate was expressed as optical density value at 450 nm (OD_450_) wavelength (n=3). (H) Flow cytometry analysis showing decreased ratios of pH3-positive fibroblasts and myofibroblasts in the group of *Safe* knockdown (n=3). (I) Representative images of immunofluorescence staining for mitosis marker 5-ethynyl-2'-deoxyuridine (EdU, magenta) and DAPI (blue), PDGFR-α (red) was stained as a marker of fibroblasts. Scale bar indicates 50 μm. Right panel: Percent of EdU^+^ cells in PDGFR-α^+^ cells (n=25). Data are presented as mean ± SEM; Student's *t*-test or one-way ANOVA; *p < 0.05.

We then investigated the function of *Safe* on cardiac fibrosis *in vitro*. Efficient suppression of *Safe* by shRNA abrogated TGF-β-induced expression of both mRNA and protein of COL1A1 and α-SMA in cardiac fibroblasts (Figure 2A-B). Immunostaining of α-SMA indicated that *Safe* shRNA blocked TGF-β-induced morphological changes in fibroblast-myofibroblast transition, as evidenced by the reduced cellular hypertrophy (cell area) and disturbed formation of stress fiber decorated by α-SMA (Figure 2C). Contractile activity is crucial for myofibroblasts to maintain the differentiated phenotype, and can be evaluated by Collagen gel contraction assay. The estimation of contraction is expressed by the area reductions of the cell-embedded collagen gels during the cell contraction period. As shown in Figure 2D, TGF-β-treated cells induced an obvious reduction of the collagen gel area when compared to the untreated fibroblasts, indicating the increased contractility of these cells. However, the contraction capacity was significantly attenuated in TGF-β-treated fibroblasts by *Safe* knockdown, which indicates reduced fibroblast-myofibroblast transition.

BMP1 is a Tolloid metalloproteinase that processes procollagen into mature collagen, the major extracellular matrix (ECM) protein deposited by myofibroblasts. In addition, BMP1 can also activate TGF-β during fibrosis. We thus assessed the effects of *Safe* on *Bmp1* mRNA expression and BMP1 activity, as well as collagen secretion into the cell culture medium. As shown in Figure 2A and Figure 2E, both the mRNA expression and enzymatic activity of BMP1 were facilitated in fibroblasts by TGF-β treatment. However, the knockdown of *Safe* significantly inhibited both basal and TGF-β-induced BMP1 expression and its activity in fibroblasts. Consistently, we observed an increased enrichment of collagen type I in culture supernatants of TGF-β-induced myofibroblasts, whereas *Safe* knockdown could mitigate the secretion of collagen type I (Figure 2F).

Because the aberrant proliferation of fibroblasts and myofibroblasts is a key event during cardiac fibrosis, we evaluated the effect of *Safe* knockdown on cell proliferation induced by TGF-β. Using the CCK-8 kit, we found that *Safe* knockdown significantly reduced the optical densities of TGF-β-treated fibroblasts on day 3 compared with control cells (Figure 2G), which implicates that *Safe* is involved in cell proliferation. We then quantified the percentages of proliferating cells by flow cytometry following pH3 staining. The percentages of pH3-positive cells were significantly lower in *Safe*-silenced cells than normal TGF-β-treated fibroblasts (Figure 2H). Furthermore, we performed double immunofluorescence staining to confirm that the EdU-positive proliferative cells stimulated by TGF-β or suppressed by *Safe* knockdown were PDGFR-α-positive fibroblasts (Figure 2I). Overall, these results indicate that TGF-β-induced cell proliferation is inhibited by *Safe* knockdown.

We then used CRISPR/Cas9-mediated knockout to further confirm the role of *Safe* in TGF-β-induced cardiac fibrosis observed above (Figure S2). The resulting data show that the knockout of *Safe* could effectively prevent cardiac fibrosis *in vitro* by inhibiting fibroblast-myofibroblast transition, aberrant cell proliferation, and secretion of ECM such as collagen type I.

### 3. Inhibition of *Safe* ameliorates MI-induced cardiac fibrosis and the impaired cardiac function

**Figure 3:**
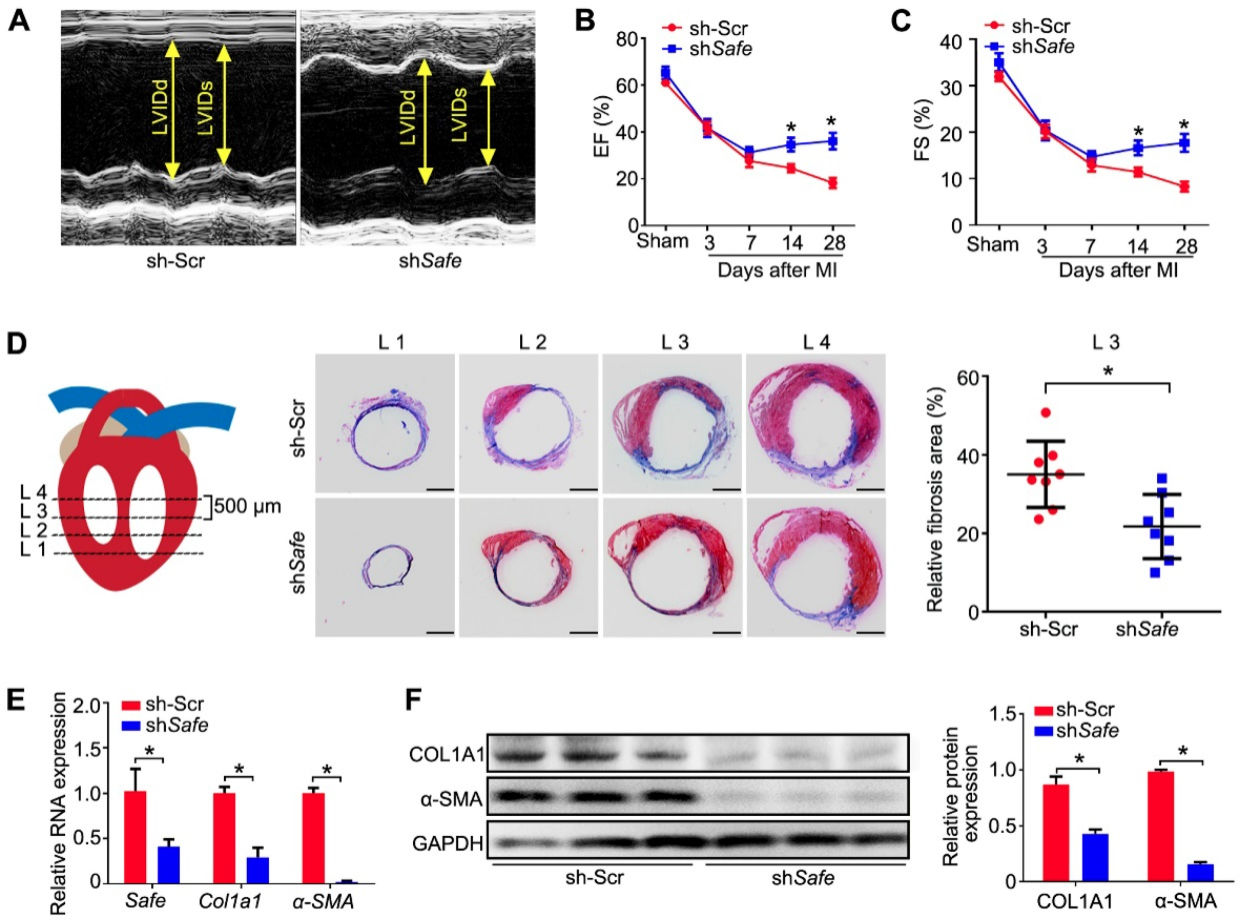
** Inhibition of *Safe* ameliorates MI-induced cardiac fibrosis.** (A) Representative M-mode echocardiographic images obtained from MI mice injected with scramble control shRNA (Ctr) or sh*Safe* lentiviral particles at day 28 after surgery (n=10 in each group). (B) Left ventricular ejection fraction (EF) of MI mice as assessed via echocardiography in indicated time points (n=10). (C) Left ventricular fractional shortening (FS) of MI hearts in indicated time points (n=10). (D) Schematic diagram of the slice, starting from left ventricular exposure, each level is 500 μm apart. Representative images of Masson's trichrome-stained MI hearts, and quantification of fibrosis (% area) showing a significant decrease of fibrosis areas in MI hearts injected with sh*Safe* lentiviral particles (n=8). Scale bar indicates 2 mm. (E) qRT-PCR analysis of *Col1a1* and *α-SMA* in the infarction zone of MI hearts (n=3). (F) Western blot analysis and relative densitometric quantification of COL1A1 and α-SMA protein levels in MI hearts after *Safe* inhibition (n=3). Data are presented as mean ± SEM; Student's *t*-test or two-way repeated-measures ANOVA; *p < 0.05.

To investigate whether the *in vivo* knockdown of *Safe* affects cardiac function in our MI model, we performed *in vivo* loss-of-function study in mice. We employed recombinant lentivirus to knock down *Safe* in mouse hearts by direct intramyocardial injection after LAD surgery. Five days post lentivirus injection, the fluorescence of GFP reporter was observed in cells around the injection sites, indicating the successful lentiviral infection (Figure S3). We found that *in vivo* inhibition of *Safe* could improve the cardiac function as evidenced by significant increases in ejection fraction (EF) and fractional shortening (FS) on days 14 and 28 post-MI (Figure 3A-C). Consistent with these results, we also observed a smaller infarction area in the injured mouse hearts after *Safe* knockdown (Figure 3D). The fibrotic markers COL1A1 and α-SMA were significantly downregulated at both mRNA and protein levels by *Safe* inhibition in MI heart (Figure 3E-F). These results therefore show that lncRNA-*Safe* plays an important role in MI-induced cardiac fibrosis and could be a potential target to improve cardiac function after myocardial infarction.

### 4.* Safe* and its neighboring gene *Sfrp2* mutually regulate each other's RNA stability in fibroblasts

**Figure 4:**
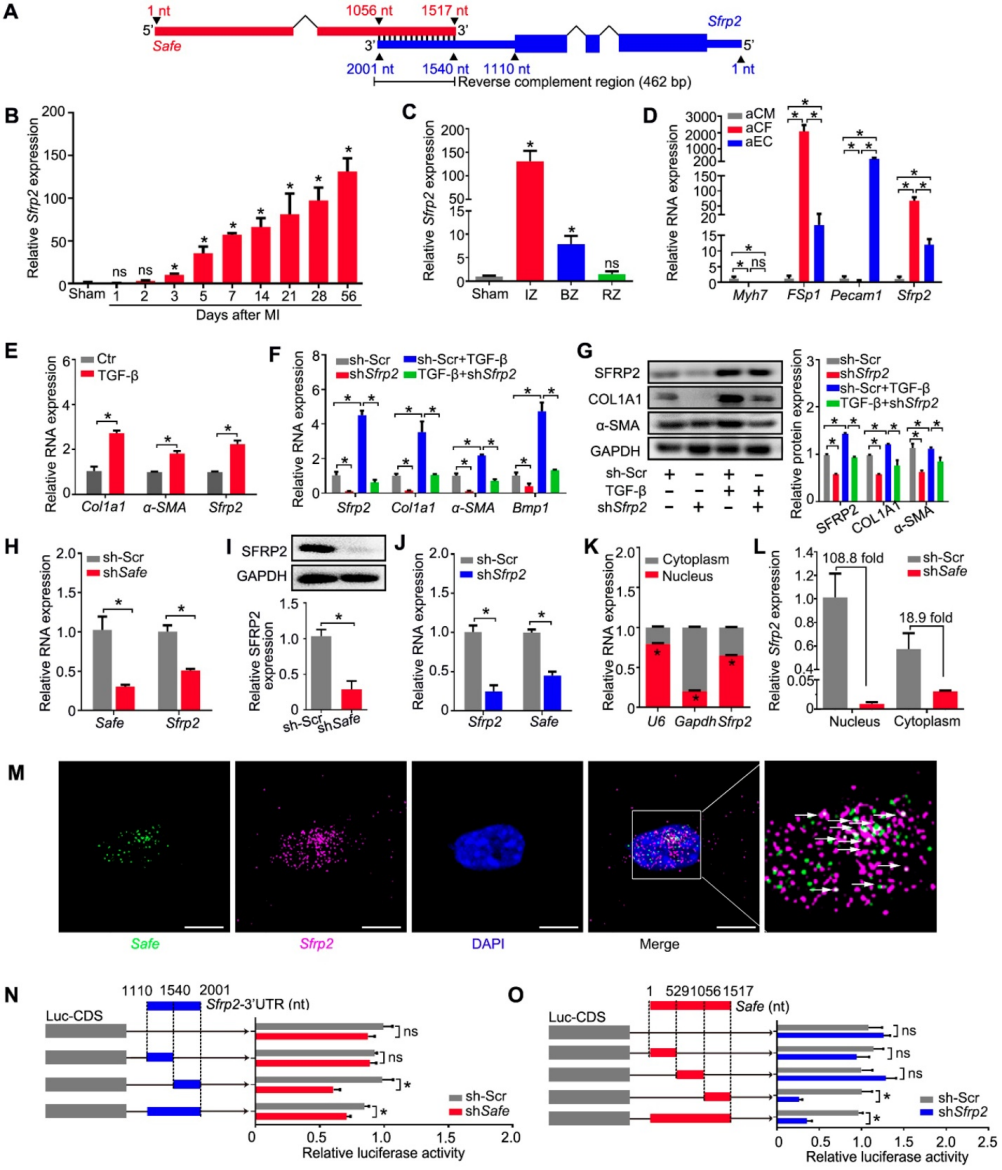
***Safe* and *Sfrp2* mutually regulates each other's RNA stability in fibroblasts.** (A) Schematic diagram of RNA-RNA interaction between *Safe* and *Sfrp2* at their reverse complementary regions (462 nucleotides in length). Thick blocks and thin blocks represent the coding sequence (CDS) and untranslated regions (UTRs), respectively. (B) qRT-PCR detection of* Sfrp2* expression in left ventricles of sham and MI mice at indicated days after surgery (n=3). (C) qRT-PCR detection of* Sfrp2* expression in the infarct zone (IZ), border zone (BZ), and remote zone (RZ) of infarcted hearts after 14 days following MI surgery (n=3). (D) qRT-PCR detection of *Sfrp2* expression in aCM, aCF and aEC (n=3). (E) qRT-PCR analysis showing increased expression of *Sfrp2* in cardiac fibroblasts after TGF-β treatment (n=3). (F) mRNA expression of *Sfrp2*, *Col1a1*, *α-SMA and Bmp1* in TGF-β-untreated or TGF-β-treated cardiac fibroblasts after *Sfrp2* knockdown (n=3). (G) Representative western blot analysis and relative densitometric quantification of SFRP2, COL1A1 and α-SMA protein levels in TGF-β-untreated or TGF-β-treated cardiac fibroblasts after *Sfrp2* knockdown (n=3). (H) qRT-PCR analysis showing decreased expression of both *Safe* and *Sfrp2* mRNA in cardiac fibroblasts transfected with sh*Safe* (n=3). (I) Representative western blot analysis and relative densitometric quantification of SFRP2 protein with *Safe* inhibition (n=)3. (J) qRT-PCR analysis showing decreased expression of both *Sfrp2* mRNA and *Safe* in cardiac fibroblasts transfected with sh*Sfrp2* (n=3). (K) Subcellular localization of *Sfrp2* in cytoplasm and nuclei of cardiac fibroblasts (n=3). (L) qRT-PCR analysis showing preferentially inhibition of *Sfrp2* mRNA level in the nucleus of cardiac fibroblasts after *Safe* knockdown (n=3). (M) Representative images of RNA FISH showing co-localization of *Safe* (green), *Sfrp2* mRNA (magenta) in the nuclei (DAPI, blue) of cardiac fibroblasts. Scale bar: 20 μm. (N) Dual luciferase assay showing sh*Safe* inhibited Firefly luciferase activities of pGL3-control vectors carrying *Sfrp2* 3'-UTR (n=3). (O) Dual luciferase assay showing sh*Sfrp2* inhibited Firefly luciferase activities of pGL3-control vectors carrying *Safe* RNA fragments complementary to *Sfrp2* mRNAs (n=3). All data are presented as mean ± SEM; Student's *t*-test or one-way ANOVA; *p < 0.05, and ns, not significant.

To identify putative target genes of *Safe*, we performed sequence analysis and found a reverse complement region (462 nt in length) of the 3'-end of *Safe* with the 3'-UTR of its nearby protein-coding gene *Sfrp2* (Figure 4A), a key regulator of cardiac fibrosis.

Our study demonstrated similar expression patterns of *Sfrp2* and *Safe* during MI-induced cardiac fibrosis. The expression of *Sfrp2* was persistently increased in the injured heart from days 3 to 56 post MI (Figure 4B), and was enriched mainly in fibrotic tissues of the infarct zone (Figure 4C). *Sfrp2* expression was much higher in fibroblasts when compared to both cardiomyocytes and endothelial cells (Figure 4D), and its expression in cardiac fibroblasts was significantly induced by TGF-β (Figure 4E). More importantly, we found that knockdown of *Sfrp2* in fibroblasts inhibited TGF-β-induced cardiac fibrosis, as evidenced by inhibited COL1A1 and α-SMA expression (Figure 4F-G), disturbed formation of α-SMA-decorated stress fiber (Figure S4A), as well as reduced cellular hypertrophy (cell area) (Figure S4A), cellular contractile activity (Figure S4B), BMP1 activity (Figure S4C), collagen type I secretion (Figure S4D) and cell proliferation (Figure S4E-G). Their similar expression patterns and functional characteristics indicated a strong correlation between *Safe* and *Sfrp2* during cardiac fibrosis.

We then assessed the effects of *Safe* and *Sfrp2* knockdown on each other's expression. Depletion of *Safe* led to a significant reduction in both SFRP2 mRNA and protein levels in primary fibroblasts (Figure 4H-I). When *Sfrp2* was inhibited by shRNA, *Safe* expression was also decreased in fibroblasts (Figure 4J). Whereas *Safe* is predominantly expressed in the nucleus, *Sfrp2* mRNA was detected in both the nucleus and cytoplasm (Figure 4K). Interestingly, nuclear *Sfrp2* expression in cardiac fibroblasts was dramatically inhibited by knockdown of *Safe*, along with a moderate decrease of cytoplasmic *Sfrp2* expression (Figure 4L). Based on the subcellular location of *Safe* and *Sfrp2* transcripts, we speculated that nuclear *Safe* and *Sfrp2* RNAs could stabilize each other via their complementary binding at the 3'-end, subsequently maintaining stable expression of SFRP2 protein.

To test our hypothesis, we firstly confirmed the nuclear localization of both *Safe* and *Sfrp2* RNAs by FISH, and found that some fluorescence signals for *Safe* were overlapped with that for *Sfrp2*, indicating the possible interaction between *Safe* and *Sfrp2* RNAs in the nuclei of fibroblasts (Figure 4M). Then we examined whether *Safe* regulates *Sfrp2* mRNA stability via the 3'-UTR of *Sfrp2* by using the pGL3-luciferase reporter system. We found that *safe* knockdown by shRNA significantly decreased the luciferase activity of pGL3 vector carrying *Sfrp2* 3'-UTR downstream the luciferase stop codon, suggesting an essential role of *Safe* transcripts on *Sfrp2* mRNA stabilization (Figure 4N). Similarly, the suppression of *Sfrp2* strongly reduced luciferase activity of pGL3 vector carrying the full-length fragment of *Safe* (Figure 4O). To identify the critical segment of *Safe* RNA and *Sfrp2* 3'-UTR responsible for the observed effect on luciferase activity, various regions of *Safe* RNA or *Sfrp2* 3'-UTR were subcloned into a pGL3-control vector for the luciferase reporter assay in NIH 3T3 cells. As illustrated in Figure 4N & 4O, the main regulatory segments in both *Safe* and *Sfrp2* 3'-UTR were located in their complementary region at the 3'-end. These data suggest that *Safe* and *Sfrp2* could promote each other's RNA stability via the RNA-RNA interaction at their complementary region.

### 5.* Sfrp2* overexpression reversed the regulatory effects of sh*Safe* in cardiac fibroblasts

**Figure 5:**
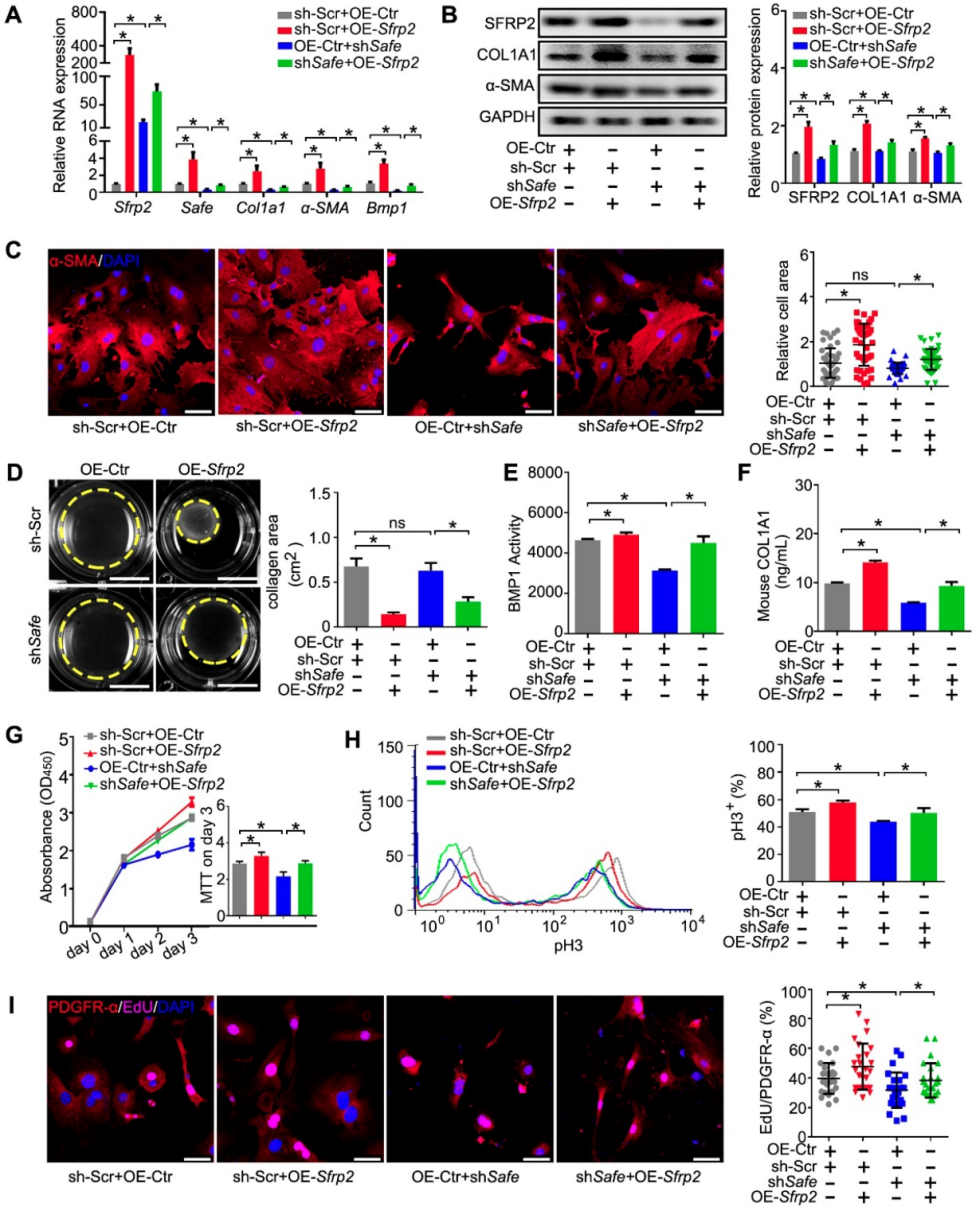
***Sfrp2* overexpression disturbed the regulatory effects of sh*Safe* in cardiac fibroblasts.** (A) qRT-PCR analysis showing mRNA expression of *Safe*, *Sfrp2, Col1a1*, *α-SMA* and *Bmp1* by *Sfrp2* overexpression in *Safe*-silenced cardiac fibroblasts (n=3). (B) Representative western blot analysis and relative densitometric quantification showing up-regulated expression of SFRP2, COL1A1, and α-SMA proteins by *Sfrp2* overexpression in *Safe*-silenced cardiac fibroblasts (n=3). (C) Representative images of immunofluorescence staining for α-SMA (red, n=5) and quantification of the relative cell area of cardiac fibroblasts after treatments as indicated (n=40). Scale bar indicates 50 μm. (D) Representative images of collagen gel contraction for 24 hours and quantification of collagen area inside the dashed circles (n=3). Scale bar indicates 0.5 cm. (E) BMP1 protein enzyme activity in the supernatant of cultured fibroblasts after indicated treatments (n=3). The excitation wavelength is 320 nm, and the emission wavelength is 405 nm. (F) ELISA assay of COL1A1 protein in the supernatant of cultured fibroblasts after indicated treatments (n=3). (G) CCK-8 assay of cardiac fibroblasts showing restored cell proliferation by *Sfrp2* overexpression in* Safe*-deficient cardiac fibroblasts (n=3). (H) Flow cytometry analysis of pH3 incorporation in *Sfrp2* overexpressed-stimulated fibroblasts with indicated treatments. (I) Representative images of immunofluorescence staining for EdU (magenta), PDGFR-α (red) and DAPI (blue). Scale bar indicates 50 μm. Right panel: Percent of EdU^+^ cells in PDGFR-α^+^ cells (n=25). All data are presented as mean ± SEM; Student's *t*-test or one-way ANOVA; *p < 0.05.

Based on the results above, we believe that *Sfrp2* could be involved in *Safe*-mediated phenotype change in cardiac fibroblasts. To test our hypothesis, we performed a rescue experiment *in vitro*. We found that *Sfrp2* overexpression significantly facilitated the expression of COL1A1, α-SMA and BMP1 in fibroblasts, and partially restored their expression in fibroblasts with* Safe* knockdown (Figure 5A-B). The average cell sizes of both normal and *Safe*-knockdown fibroblasts were significantly increased by* Sfrp2* overexpression (Figure 5C). Furthermore, *Sfrp2* overexpression significantly increased the contraction capacity of both normal and *Safe*-knockdown fibroblasts, as indicated by the reduced collagen gel area (Figure 5D). Although *Safe* knockdown dramatically inhibited BMP1 activity and COL1A1 secretion from fibroblasts, the reduced BMP1 activity and COL1A1 content in cell supernatant was partially rescued by* Sfrp2* overexpression (Figure 5E-F). Cell proliferation analyses also indicated a reversed role of *Sfrp2* overexpression in sh*Safe*-mediated suppression of cell proliferation (Figure 5G-I). Taken together, our results indicate that *Sfrp2* overexpression disturbs the regulatory effect of sh*Safe* in cardiac fibroblasts.

### 6.* Sfrp2* overexpression abrogated sh*Safe*-mediated improvement of heart function post MI

**Figure 6:**
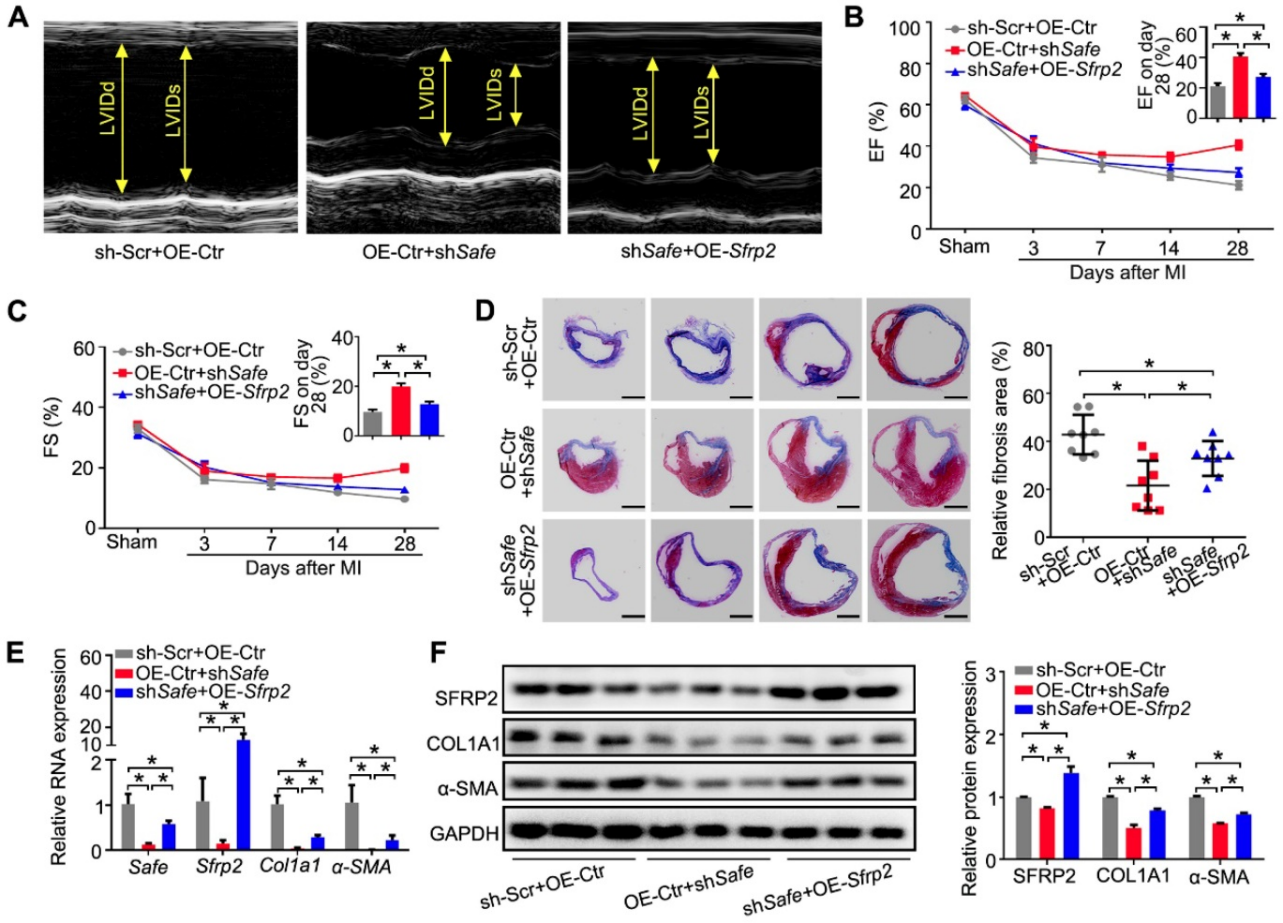
***Sfrp2* overexpression disturbed the protective effect of sh*Safe* on cardiac function post MI*.*** (A) Representative M-mode echocardiographic images obtained from MI mice after *Safe* knockdown, in combination with *Sfrp2* overexpression (n=10 in each group). (B) *Sfrp2* overexpression led to a reduced improvement of EF in sh*Safe*-injected MI mice at day 28 post-surgery (n=10). (C) sh*Safe*-mediated increase in FS in MI hearts was inhibited by *Sfrp2* overexpression (n=10). (D) Representative images of Masson's trichrome-stained MI hearts, and quantification of relative fibrosis areas (n=8). Scale bar indicates 2 mm. (E) qRT-PCR analysis showing increased expression of *Safe, Sfrp2*, *Col1a1* and *α-SMA* in left ventricles of sh*Safe*-injected MI hearts after *Sfrp2* overexpression (n=3). (F) Western blot analysis and relative densitometric quantification of SFRP2, COL1A1 and α-SMA protein levels in sh*Safe*-injected MI hearts after *Sfrp2* overexpression (n=3). All data are presented as mean ± SEM; Student's *t*-test, one-way ANOVA or two-way repeated-measures ANOVA, *p < 0.05.

We then tested whether *Sfrp2* can regulate *Safe*-mediated cardiac fibrosis post MI. The lentivirus-mediated *Safe* knockdown or *Sfrp2* overexpression at the infarction sites was implemented through intramyocardial injection of corresponding lentiviral particles immediately post MI induction. Echocardiographic data indicated a reverse effect of *Sfrp2* overexpression on cardiac functional improvement induced by *Safe* shRNAs. On day 28 post-MI, we observed dramatic increases in EF and FS in sh*Safe*-injected mice when compared with the control group. However, *Sfrp2* overexpression resulted in significant declines of both EF and FS when compared with the sh*Safe* group (Figure 6A-C). The areas of fibrosis in the infarcted hearts were then assessed following Masson's trichrome staining on 4 successive 500 μm planes. Despite the obvious decrease of fibrosis area in sh*Safe*-injected MI hearts, sh*Safe*-induced reduction of cardiac fibrosis was significantly prevented by lentivirus-mediated *Sfrp2* overexpression (Figure 6D). In line with this, the impaired expressions of fibrotic markers COL1A1 and α-SMA in sh*Safe*-treated MI hearts were also partially reversed by *Sfrp2* overexpression, as indicated by qRT-PCR and western blot results (Figure 6E-F). These data indicate that *Sfrp2* plays a significant role in *Safe*-mediated cardiac fibrosis.

### 7. Binding of HuR to the *Safe*-*Sfrp2* duplex safeguards RNA stabilization of both *Safe* and *Sfrp2*

Since *Safe* and *Sfrp2* RNAs promote each other's RNA stability, we intended to identify putative proteins binding to their complementary region. A total of 10 RNA-binding proteins were predicated to bind to the complementary region of *Safe* and *Sfrp2* RNAs by the Database of RNA-Binding Protein Specificities (RBPDB, http://rbpdb.ccbr.utoronto.ca/). The predicted protein candidates include PABPC1, ELAVL1 (also known as human antigen R, HuR), EIF4B, PUM2, RBMX, SFRS1, ZRANB2, RBMY1A1, A2BP1, and MBNL1. Among them, the RNA-binding protein HuR, whose binding sites distribute in 5 different loci (5'-GUUU-3') of the complementary region of *Safe*, has been involved in TGF-β-induced fibrosis by modulating target RNA stability. We thus speculated that the HuR protein may regulate cardiac fibrosis by promoting complementary binding of *Safe* and *Sfrp2*.

Compared with *Safe* and *Sfrp2*, HuR was expressed in a similar pattern in both healthy and diseased hearts. The expression of *HuR* was significantly increased in the injured hearts on days 14-56 post MI, and was primarily enriched in the infarcted tissues Figure S5A-B). In adult mouse hearts (Figure S5C), *HuR* was mainly expressed in fibroblasts rather than cardiomyocytes, and its protein was primarily localized in the nuclei of cardiac fibroblasts (Figure S5D-E). Meanwhile, *HuR* expression in fibroblasts was significantly elevated by TGF-β (Figure S5F).

**Figure 7:**
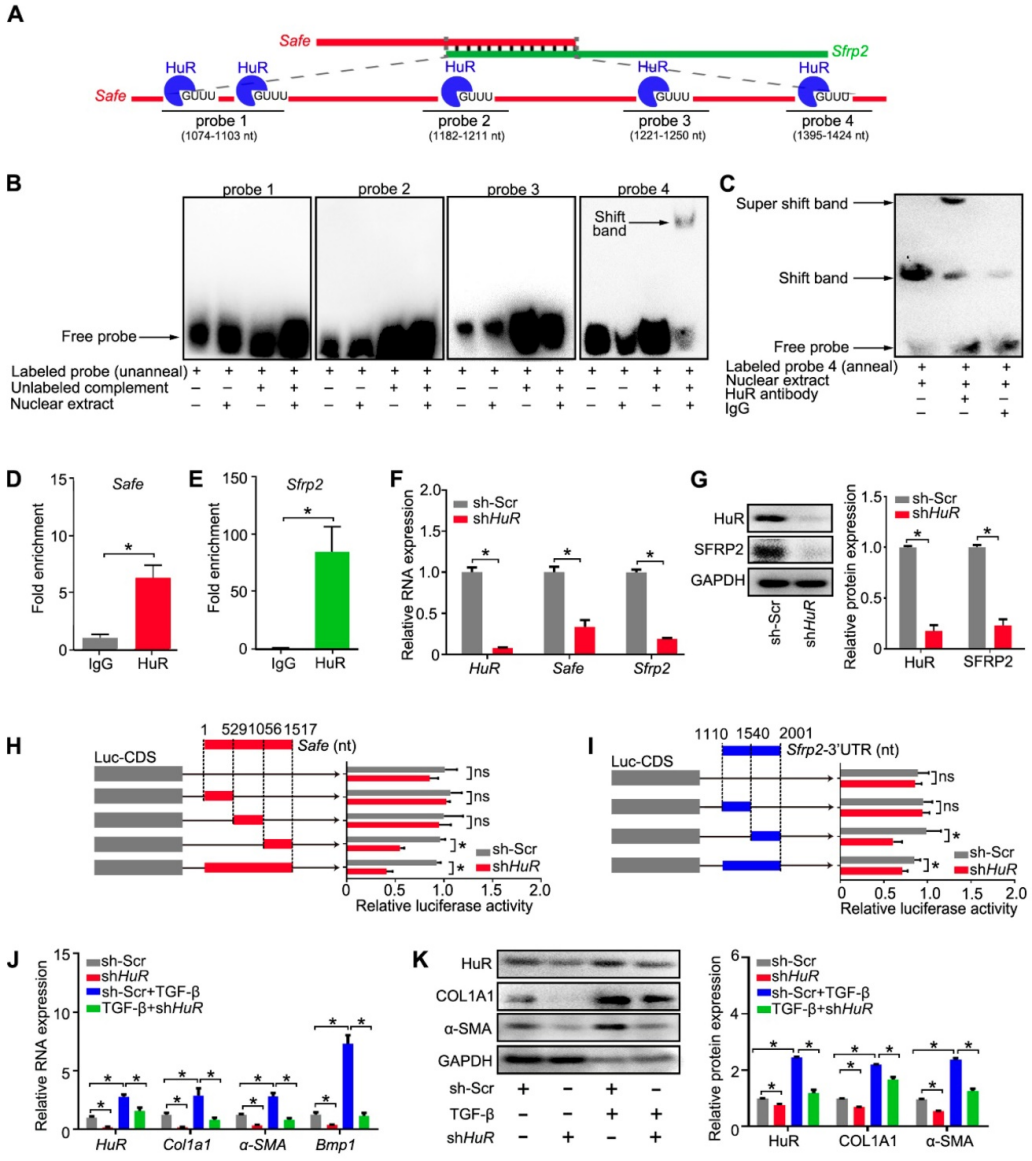
** Binding of HuR to the *Safe*-*Sfrp2* duplex accelerates RNA stabilization of both *Safe* and *Sfrp2*.** (A) Schematic presentation of predicated HuR binding sites and corresponding RNA probes for EMSA assay in the complementary region of *Safe* and *Sfrp2* RNA. (B) EMSA assay indicating specific binding of nuclear proteins of cardiac fibroblasts to the 26-nucleotide RNA duplexes corresponding to nucleotide at the 1408 nt protein binding site of *Safe*. (C) EMSA supershift assay revealing the *in vitro* interaction between HuR protein and the 26-nucleotide RNA duplexes in nuclear extracts. RNA immunoprecipitation (RIP) assay using HuR antibody and IgG (isotype control) showing enrichment of *Safe* (D) and *Sfrp2* (E) RNAs to HuR protein. (F) qRT-PCR analysis showing decreased expression of *HuR*, *Safe* and *Sfrp2* in cardiac fibroblasts after *HuR* knockdown (n=3). (G) Representative western blot analysis and relative densitometric quantification of HuR and SRRP2 protein levels in cardiac fibroblasts with or without *HuR* inhibition (n=3). (H) Dual luciferase assay showing sh*HuR* inhibited Firefly luciferase activities of pGL3-control vectors carrying the 3'-end of *Safe* (462-nucleotide in length) (n=3). (I) Dual luciferase assay showing sh*HuR* inhibited Firefly luciferase activities of pGL3-control vectors carrying *Sfrp2* 3'-UTR (n=3). (J) qRT-PCR detection of *HuR*, *Col1a1*, *α-SMA and Bmp1* in TGF-β-untreated and TGF-β-treated cardiac fibroblasts after *HuR* knockdown (n=3). (K) Representative western blot analysis and relative densitometric quantification of HuR, COL1A1 and α-SMA protein levels in sh-Scr or *HuR*-silenced cardiac fibroblasts after TGF-β treatment (n=3). All data are presented as mean ± SEM; Student's *t*-test or one-way ANOVA, *p < 0.05, and ns, not significant.

To demonstrate the direct interaction between HuR and* Safe*-*Sfrp2* duplex, we performed RNA EMSA with the nuclear component of fibroblasts. As shown in Figure 7A, four biotin-labeled RNA probes were synthesized, including one probe flanking two adjacent HuR binding sites. The nuclear extracts from fibroblasts were incubated with these probes, either individually or in combination with their unlabeled complementary fragments, followed by electrophoresis of the pull-down complexes. No obvious shift band was detected when these probes were individually incubated with the nuclear extracts (Figure 7B). After being annealed with their unlabeled complementary RNA, only the 4^th^ probe (1395-1424nt) of *Safe*, corresponding to the 1408nt binding site, displayed a stable interaction with proteins from the nucleus (Figure 7B), meanwhile, the HuR antibody successfully shifted the RNA-protein complex (Figure 7C). These data indicate that nuclear HuR could recognize the GU-rich element at 1408 nt binding site of *Safe* and bind to the* Safe*-*Sfrp2* RNA duplex.

Consistent with EMSA results, the RNA immunoprecipitation (RIP) assay revealed that both *Safe* and *Sfrp2* were enriched in HuR immunoprecipitation samples when compared to samples immunoprecipitated with the control IgG (Figure 7D-E). In addition, the expression levels of *Safe* and *Sfrp2* were significantly decreased in fibroblast transfected with *HuR* shRNA (Figure 7F-G). Using the luciferase reporter assays, we found that knockdown of *HuR* dramatically inhibited luciferase activity of pGL3 vectors carrying 3'-UTR of *Sfrp2*, a full-length fragment of *Safe* or their complemental 3'-end fragments (Figure 7H-I). These results indicate that HuR protein safeguards *Safe* and *Sfrp2* expression by binding to their complementary RNA duplex.

Given its contribution in *Safe* and *Sfrp2* stabilization, we then evaluated the role of HuR in cardiac fibrosis *in vitro*. Knockdown of *HuR* reduced the levels of COL1A1, α-SMA and BMP-1 mRNA and protein in cardiac fibroblasts (Figure 7J-K), and prevented TGF-β-induced changes of cell morphology (Figure S5G), contraction capacity (Figure S5H), BMP-1 activity (Figure S5I), COL1A1 secretion (Figure S5J), and cell proliferation (Figure S5K-M). Taken together, these data indicate that HuR can bind to *Safe*-*Sfrp2* duplex and regulate cardiac fibrosis.

We further investigated whether *Safe* overexpression could resist the regulatory effects of *Sfrp2* or *HuR* knockdown in cardiac fibroblasts. As shown in Figure S6, *Safe* overexpression could partially restore the reduced expression of fibrosis-related genes including *Sfrp2*, *Col1a1*, *α-SMA* and *Bmp1* in *Sfrp2*-knockdowned fibroblasts, and prevent the cell phenotype changes induced by *Sfrp2* shRNAs. However, the* Safe* overexpression failed to obviously rescue the reduced fibrosis-related gene expression, as well as the impaired fibrotic phenotype, in *HuR*-silenced fibroblasts (Figure S7). These data indicated a critical role of the RNA-binding protein HuR in protection of *Safe*-mediated *Sfrp2* mRNA stabilization and subsequent events of cardiac fibrosis.

## Discussion

In this study, we uncovered 389 differentially-expressed lncRNAs in mouse fibrous tissue compared to cardiac tissue from remote regions. Although previous studies have revealed beneficial effects of several lncRNAs on pathological fibrosis, most of them were cardiomyocyte-enrichment 19-21. *Wisper*, *Meg3*, and *MIAT* are three of the few known cardiac lncRNAs showing direct contribution to cardiac fibrosis 13-15. Here, lncRNA-*Safe* (*AK137033*) was shown to be highly expressed in fibroblasts in comparison with cardiomyocytes and endothelial cells from mouse hearts, and its expression was continuously increased in the fibrous tissue after myocardial infarction. Despite its potential significance in cardiac fibrosis, no experimental studies on the specific role of *Safe* and the mechanisms of action have been conducted until now. Here, we provided the first evidence that lncRNA-*Safe* is a critical regulator of cardiac fibrosis. Suppression of *Safe* could inhibit both *in vitro* TGF-β-induced cardiac fibrosis and *in vivo* MI-induced cardiac fibrosis, and improve cardiac function after myocardial infarction.

The fibrotic response after MI can be classified into two types of fibrosis, namely replacement fibrosis occurring at the infarct zone, and reactive fibrosis occurring at the border zone or remote zone 22. According our microarray and qPCR data, the expression levels of *Safe* was significantly increased in cells from the infarct zone when compared to that in the border zone and remote zone sample. These data indicate *Safe* is primarily involved in the replacement fibrosis post MI. Compared to MI-model, the TAC model is much more suitable to study the reactive fibrosis. We thus focus on the effect of *Safe* on the replacement fibrosis in this study. However, *Safe* may be also involved in MI-induced reactive fibrosis, since we also observed a moderately increased expression of *Safe* in the border zone when compared to that in the sham group. Moreover, both replacement fibrosis and reactive fibrosis are mediated by fibroblasts and myofibroblasts in similar mechanisms 22.

Cardiac fibrosis is caused by stress-induced activation of fibroblast proliferation and fibroblast-to-myofibroblast transition, and characterized by excessive deposition of fibrous extracellular matrix (ECM) by myofibroblasts in the heart 23. TGF-β has been regarded as master regulator of fibrosis in many organs 24. Our *in vitro* study showed that *Safe* knockdown could prevent TGF-β-induced cardiac fibrosis, via the inhibition of fibroblast proliferation, fibroblast-myofibroblast transition, and subsequent secretion of collagen type I. These data indicate that *Safe* is a critical mediator of TGF-β signaling in the process of cardiac fibrosis. Consistent with *in vitro* observations, the inhibition of *Safe* significantly abolished MI-induced cardiac fibrosis and improved the heart function. Although TGF-β signaling has been considered as a promising therapeutic target for cardiac fibrosis, efforts to translate this concept have been hampered by concerns about potential adverse effects of targeting TGF‑β itself due to the complex pleiotropic effects of TGF-β signaling in many biological responses 24-26. Our study therefore provides a promising alternative therapeutic strategy to inhibit cardiac fibrosis by targeting the lncRNA-*Safe* that underlies TGF‑β‑induced fibrosis.

LncRNAs have been proposed to carry out diverse functions that are intimately dependent on their locations in the cell 27, 28. Whereas nuclear lncRNAs are mainly involved in gene transcription and chromatin organization, cytoplasmic lncRNAs regulate mRNA stability and translation. For instance, nuclear lncRNA *Meg3* in fibroblasts regulates the transcription of matrix metalloproteinase-2 (*Mmp2*) in cardiac fibroblasts through recruiting P53 onto *Mmp2* promoter 13. Despite its nuclear expression, we found that* Safe* can promote RNA stability of its neighboring protein-coding gene *Sfrp2*, and vice versa, through the formation of complementary RNA duplex at their 3'-end. Knockdown of *Safe* dramatically inhibited mRNA abundancy of *Sfrp2* in the nucleus, but only moderately reduced its expression in the cytoplasm. Consistent with our data, recent studies reported the role of nucleus-enriched mRNAs in buffering cytoplasmic transcript levels from drastic change 29. Thus, the formation of *Safe*-*Sfrp2* duplex in nucleus might promote the protection role of nuclear *Sfrp2* mRNA in cytoplasmic *Sfrp2* mRNA and protein expression. Despite the reported biphasic effects of *Sfrp2* on cardiac fibrosis, our studies revealed a protective role of *Sfrp2* knockdown in TGF‑β-induced fibrosis, consistent with previous studies in *Sfrp2*-deficient mice 30. Most importantly, *Sfrp2* overexpression disturbed the regulatory effect of sh*Safe* in both *in vitro* cultured cardiac fibroblasts and MI-induced fibrosis. These data provide strong evidences for the primary role of *Safe*-*Sfrp2* duplex in cardiac fibrosis.

HuR has been recently described as a contributor of various types of fibrosis by stabilizing target RNAs by binding GU-rich or AU-rich elements in their 3'-UTRs 31-34. Knockdown of *HuR* represses MI-induced cardiac fibrosis, left ventricle dysfunction, and remodeling 31, 35. Consistent with these *in vivo* studies, our study shows an inhibitory role of *HuR* knockdown in TGF‑β‑induced cardiac fibrosis *in vitro*, a similar phenotype of *Safe* or *Sfrp2* deficiencies. The known mechanisms of HuR action on MI-induced events include increasing TNF-α-associated inflammatory cell infiltration, promoting p53-associated myocardial apoptosis, and inducing TGF‑β-associated cardiac fibrosis by direct binding and stabilizing their mRNAs 35. In this study, we present a novel mechanism of HuR action that HuR protein binds to *Safe*-*Sfrp2* RNA duplex and stabilizes both *Safe* and *Sfrp2* in cardiac fibrosis. Due to the pro-fibrotic function of each gene, we believe that HuR-mediated stabilization of *Safe*-*Sfrp2* RNA duplex may play a pivotal role in both MI and TGF‑β‑induced cardiac fibrosis.

**Figure 8:**
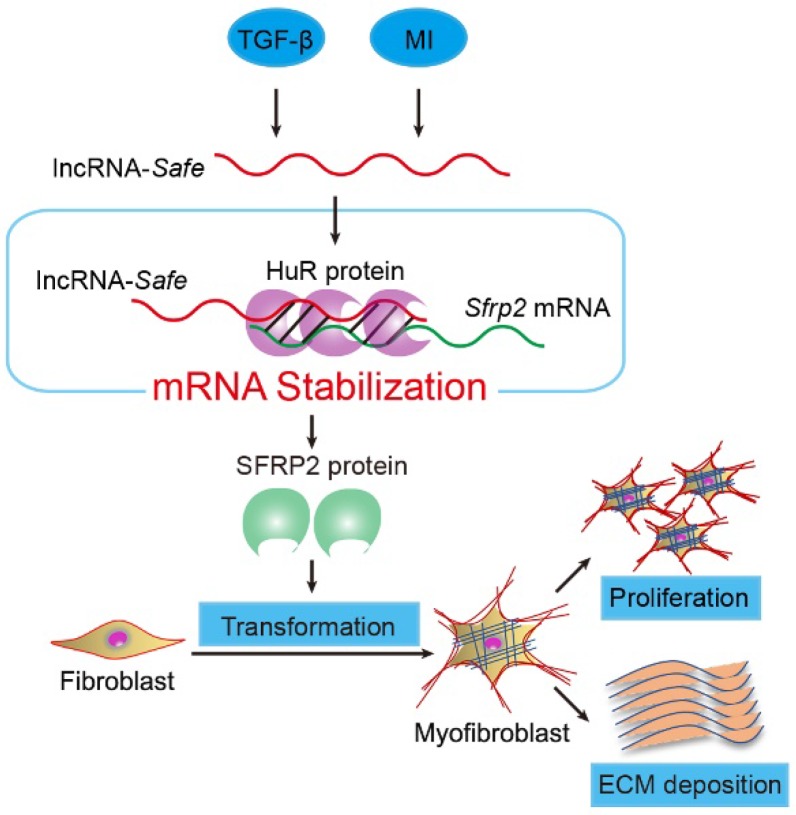
** Mechanism of lncRNA-*Safe*-mediated cardiac fibrosis.** Pathological stimuli such as TGF-β and myocardial infarction facilitate the expression of lncRNA-*Safe* in cardiac fibroblasts. In cooperation with HuR protein, *Safe* can complementarily binds to the 3'-UTR of *Sfrp2* mRNA, and promote *Sfrp2* RNA stabilization and protein expression, which in turn accelerates the transformation and proliferation of myofibroblasts, eventually leading to excessive secretion of extracellular matrix proteins.

In summary, our data demonstrate that lncRNA-*Safe* plays a critical role in cardiac fibrosis, at least partially via its ability to promote *Safe*-*Sfrp2*-HuR complex-mediated* Sfrp2* mRNA stability and protein expression (Figure 8). Inhibition of *Safe* could prevent TGF‑β-induced activation of fibroblast proliferation, fibroblast-myofibroblast transition, and collagen secretion, thus restraining cardiac fibrosis and improving cardiac function after myocardial infarction. Our results are the first to show that fibroblast-expressed lncRNA-*Safe* may function as a novel target for future anti-fibrotic therapy in heart disease.

## Supplementary Material

Supplementary figures and tables.
