# Supplementary material for: LncRNA-Safe contributes to cardiac fibrosis through Safe-Sfrp2-HuR complex in mouse myocardial infarction
Source: Theranostics. 2019 Sep 25;9(24):7282–97. doi: 10.7150/thno.33920 (PMC6831303; doi:10.7150/thno.33920)
Supplement: Supplementary file 1 — Supplementary figures and tables. [file thnov09p7282s1.pdf]

SUPPLEMENTAL INFORMATION

LncRNA-*Safe* contributes to cardiac fibrosis through *Safe*-*Sfrp2*-HuR complex in mouse myocardial infarction

Kaili Hao[1, #], Wei Lei[1, #], Hongchun Wu[1], Jie Wu[1], Zhuangzhuang Yang[1], Shiping Yan[1], Xing-Ai Lu[1], Jingjing Li[1], Xue Xia[1], Xinglong Han[1], Wenbo Deng[2], Guisheng Zhong[3], Zhen-Ao Zhao[1], Shijun Hu[1, *]

[1]Department of Cardiovascular Surgery of the First Affiliated Hospital & Institute for Cardiovascular Science, State Key Laboratory of Radiation Medicine and Protection, Medical College, Soochow University, Suzhou 215000, China; [2]Fujian Provincial Key Laboratory of Reproductive Health Research, School of Medicine, Xiamen University, Xiamen 361102, China; [3]iHuman Institute, School of Life Science and Technology, ShanghaiTech University, Shanghai 210021, China.

[#] Co-first authors

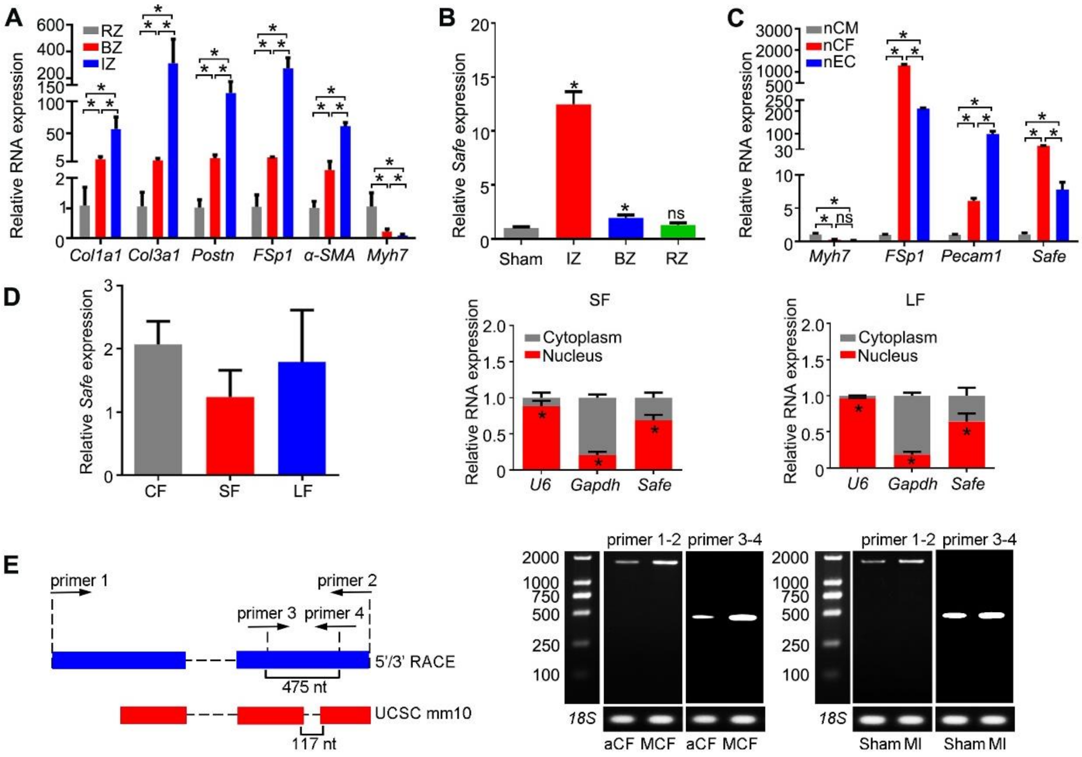

Figure S1 ***Safe*** **distribution in the nucleus of fibroblasts.** (A) qRT-PCR detection of *Col1a1*, *Col3a1*, *Postn*, *FSp1*, *α-SMA* and *Mhy7* in infarct zone (IZ), border zone (BZ) and remote zone (RZ) of MI hearts at day 14 post MI (n=3). (B) qRT-PCR detection of *Safe* expression in IZ, BZ and RZ of MI hearts at day 14 post MI (n=3). (C) qRT-PCR detection of *Safe* expression in cardiomyocytes (nCM), cardiac fibroblasts (nCF) and endothelial cells (nEC) from neonatal mouse hearts (n=3). (D) qRT-PCR detection of *Safe* expression and subcellular localization in cardiac fibroblasts (CF), skin fibroblasts (SF) and lung fibroblasts (LF) (n=3). (E) Schematic diagram of identifying whether there were different variants of *Safe*. Primer 1-2 (1 bp-1517 bp) were used to identify the full length determined by RACE, primer 3-4 (938 bp-1412 bp) was used to identify the different variants. Right: Agarose gel electrophoresis of the PCR products amplified from cardiac fibroblasts (CF) and myofibroblasts (MF), as well as in sham-operation (Sham) and myocardial infarction (MI) cardiac samples. All data are presented as mean ± SEM; Student's *t*-test or one-way ANOVA; *$p < 0.05$, and ns, not significant.

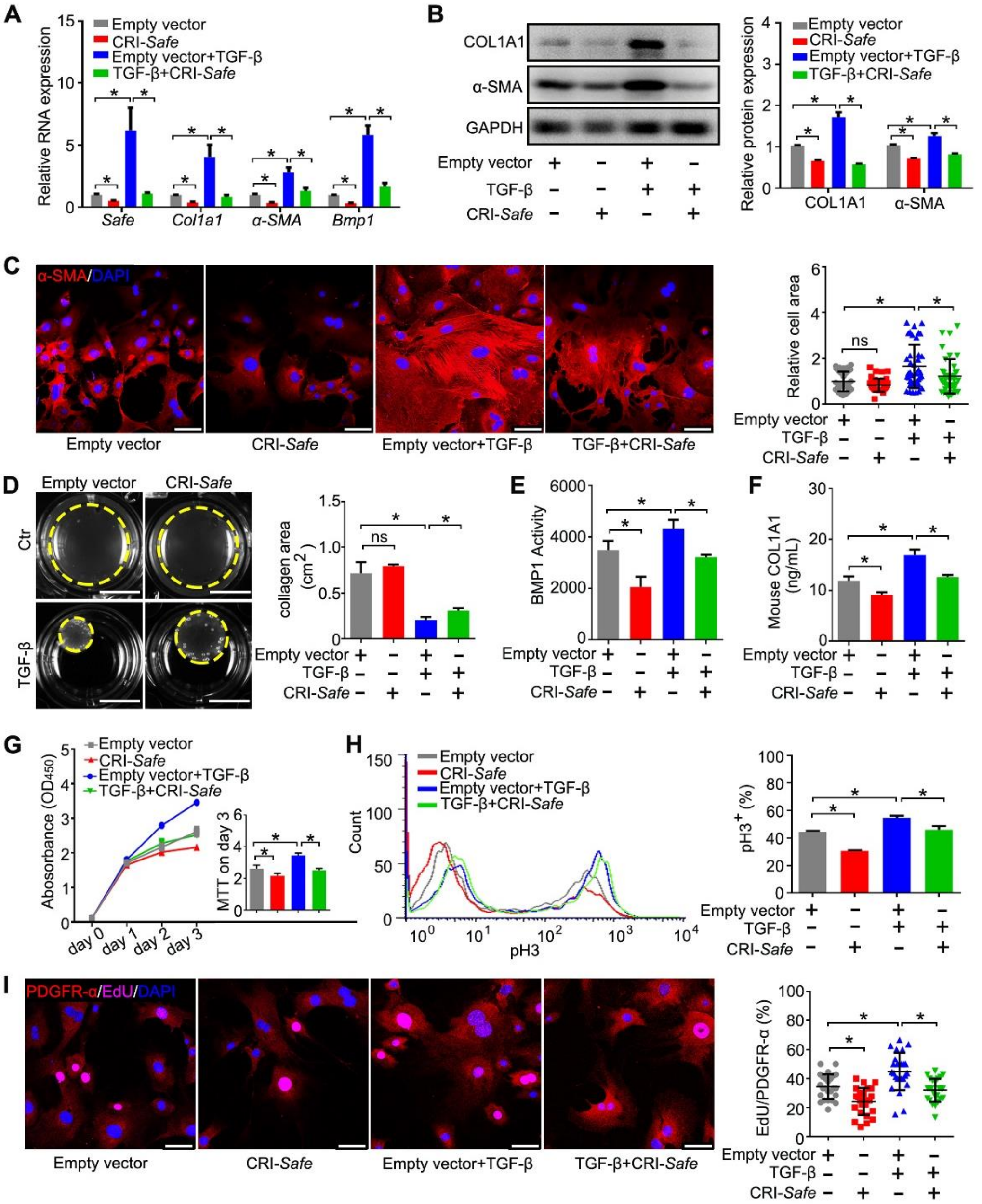

Figure S2 CRISPR/Cas9-mediated *Saf*e inhibition prevents TGF-β-induced cardiac fibrosis. (A) qRT-PCR analysis showing inhibition of *Safe*, *Col1a1*, *α-SMA* and *Bmp1* expression in TGF-β-untreated or TGF-β-treated cardiac fibroblasts by CRISPR/Cas9-mediated *Safe* silence (n=3). (B) Representative western blot analysis and relative densitometric quantification of COL1A1 and α-SMA protein levels in TGF-β-untreated or TGF-β-treated cardiac fibroblasts after CRISPR/Cas9-mediated *Safe* inhibition (n=3). (C) Representative images of immunofluorescence staining for α-SMA (red, n=5) and quantification of the relative cell area of cardiac fibroblasts after treatments as indicated (n=40). Scale bar indicates 50 μm. (D) Representative images of collagen gel contraction for 24 hours and quantification of collagen area inside the dashed circles. (E) BMP1 protein enzyme activity in the supernatant of cultured fibroblasts

after indicated treatments. The excitation wavelength is 320 nm, the emission wavelength is 405 nm (n=3). (F) ELISA assay of COL1A1 protein in the supernatant of cultured fibroblasts after indicated treatments. (G) CCK-8 assay of cardiac fibroblasts showing repressed cell proliferation by CRISPR/Cas9-mediated *Safe* inhibition. (H) Flow cytometry analysis showing the ratio of pH3-positive cells was reduced by *Safe* inhibition (n=3). (I) Representative images of immunofluorescence staining and relative densitometric quantification for mitosis marker 5-ethynyl-2'-deoxyuridine (EdU, magenta) and DAPI (blue), PDGFR-α (red) was stained as a marker of fibroblasts. Scale bar indicates 50 μm. Right panel: Percent of EdU^+ cells in PDGFR-α^+ cells (n=25). All data are presented as mean ± SEM; Student's *t*-test or one-way ANOVA; $*p < 0.05$.

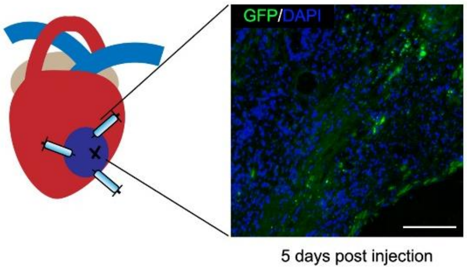

Figure S3 Schematic diagram of lentivirus injection into the heart. The lentiviral particles carrying GFP were injected into infarct area at three points. The detection of GFP-positive cells indicates successful lentiviral infection.

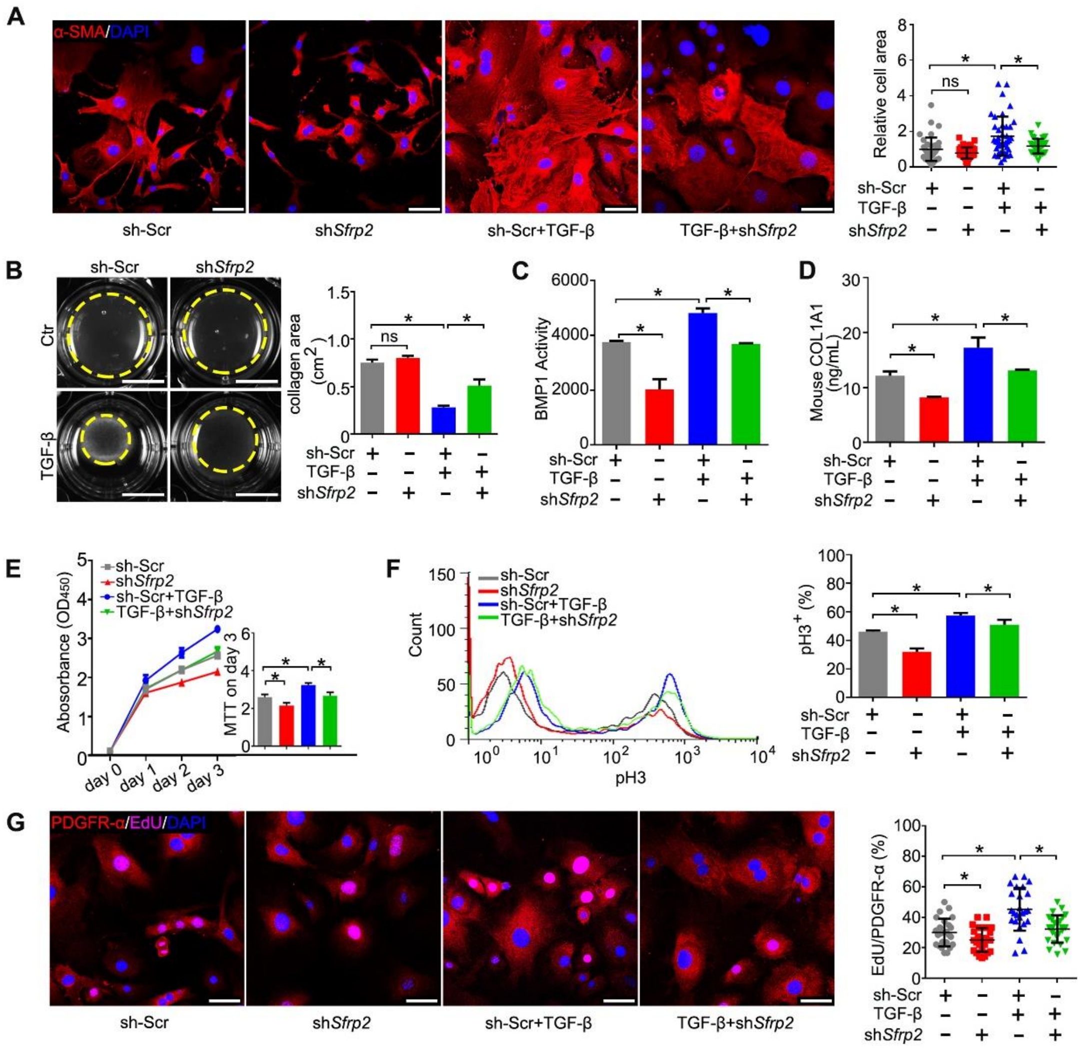

Figure S4 *Sfrp2* knockdown restrains TGF-β-induced cardiac fibrosis *in vitro*. (A) Representative images of immunofluorescence staining for α-SMA (red) and DAPI (blue), and quantification of the relative cell area of cardiac fibroblasts (n=40). Scale bar indicates 50 μm. (B) Representative image of collagen gel contraction for 24 hours and quantification of collagen area inside the dashed circles (n=3). Scale bar indicates 0.5 cm. (C) BMP1 protein enzyme activity in the supernatant of cultured fibroblasts after indicated treatments (n=3). The excitation wavelength is 320 nm, while the emission wavelength is 405 nm. (D) ELISA assay of COL1A1 protein in the supernatant of cultured fibroblasts after indicated treatments. (E) CCK-8 assay of cardiac fibroblasts showing repressed cell proliferation by *Sfrp2* inhibition during TGF-β-induced cardiac fibrosis. The cell proliferation rate was expressed as optical density value at 450 nm (OD_{450}) wavelength. (F) Flow cytometry analysis showing the ratio of pH3-positive cells was reduced by *Sfrp2* inhibition (n=3). (G) Representative images of immunofluorescence staining and relative densitometric quantification for mitosis marker 5-ethynyl-2'-deoxyuridine (EdU, magenta) and DAPI (blue), PDGFR-α (red) was stained as a marker of

fibroblasts. Scale bar indicates 50 μm. Right panel: Percent of EdU^+ cells in PDGFR-α^+ cells (n=25). All data are presented as mean ± SEM; Student's *t*-test or one-way ANOVA; *$p < 0.05$.

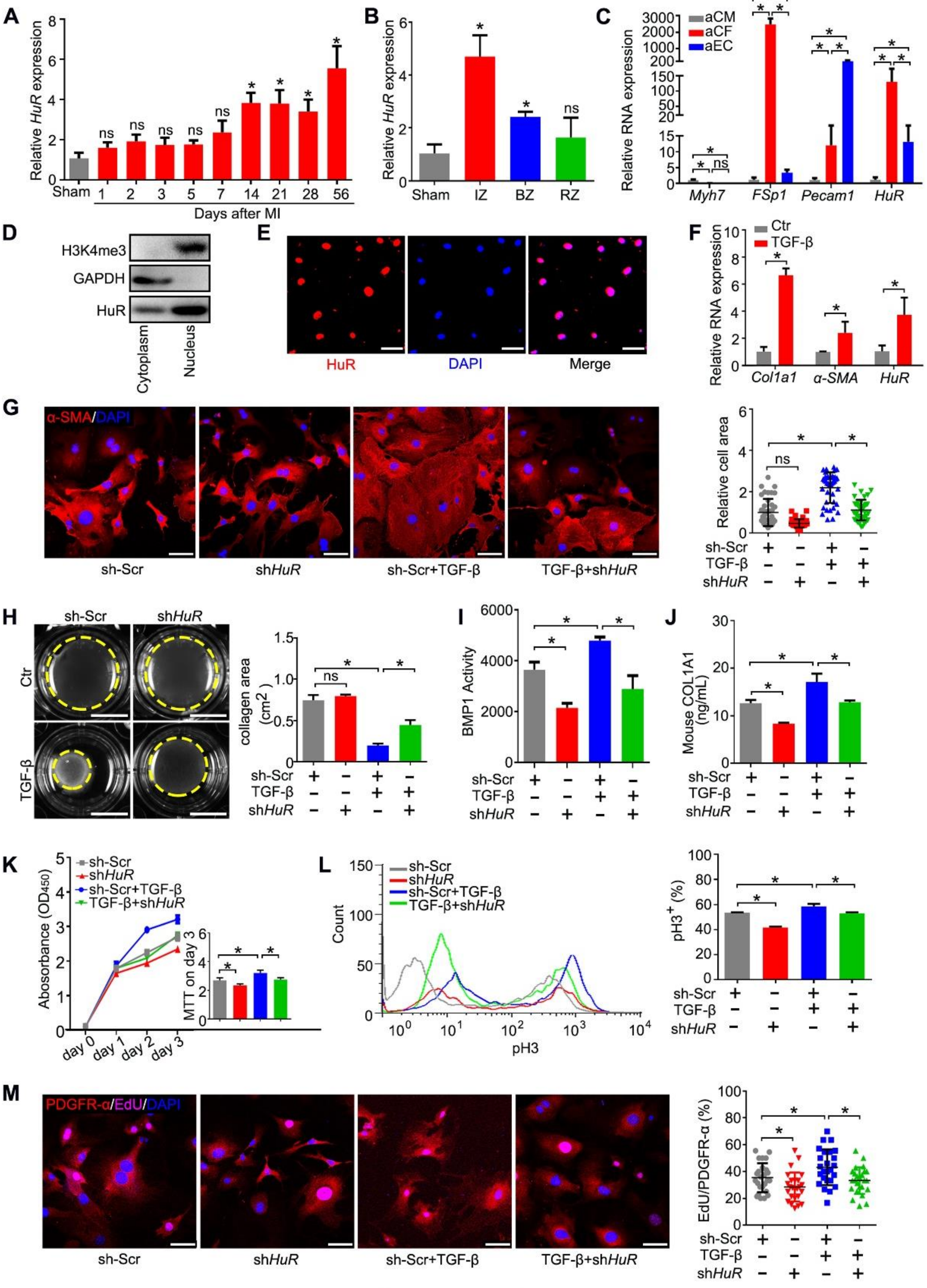

Figure S5 HuR is involved in MI and TGF-β-induced cardiac fibrosis. (A) qRT-PCR analysis of *HuR* expression in left ventricles of sham or MI hearts at indicated days post MI (n=3). (B) qRT-PCR detection of *HuR* expression in the infarct zone (IZ), border zone (BZ) and remote zone (RZ) of MI hearts at day 14

post-surgery (n=3). (C) qRT-PCR detection of *HuR* expression level in aCM, aCF and aEC (n=3). Representative images of western blot (D) and immunofluorescence staining (E) for HuR showing the nuclear localization of HuR in cardiac fibroblasts. The protein H3K4me3 and GAPDH were used as nuclear and cytosol protein markers, respectively. DAPI (blue) stained the nucleus. (F) qRT-PCR analysis showing increased expression of *HuR* mRNA in cardiac fibroblasts after TGF-β treatment (n=3). (G) Representative images of immunofluorescence staining for α-SMA (red) and DAPI (blue) in TGF-β-treated cardiac fibroblasts (n=5). Scale bar indicates 50 μm. Right panel: quantification of the relative cell area of cardiac fibroblasts (n=40). (H) Representative images of collagen gel contraction. Right panel: quantification of collagen area inside the dashed circles (n=3). Scale bar indicates 0.5 cm. (I) BMP1 protein enzyme activity in the supernatant of cultured fibroblasts after indicated treatments (n=3). The excitation wavelength is 320 nm, and the emission wavelength is 405 nm. (J) ELISA assay of secreted COL1A1 protein from TGF-β-treated fibroblasts with or without *HuR* inhibition. (K) CCK-8 assay of cardiac fibroblasts showing repressed cell proliferation by *HuR* inhibition during TGF-β-induced cardiac fibrosis. (L) Flow cytometry analysis showing the ratio of pH3-positive cells was reduced by *HuR* inhibition (n=3). (M) Representative images of immunofluorescence staining and relative densitometric quantification for mitosis marker 5-ethynyl-2'-deoxyuridine (EdU, magenta) and DAPI (blue), PDGFR-α (red) was stained as a marker of fibroblasts (n=4). Scale bar indicates 50 μm. Right panel: Percent of EdU^+ cells in PDGFR-α^+ cells (n=25). All data are presented as mean ± SEM; Student's *t*-test or one-way ANOVA; $*p < 0.05$ and ns, not significant.

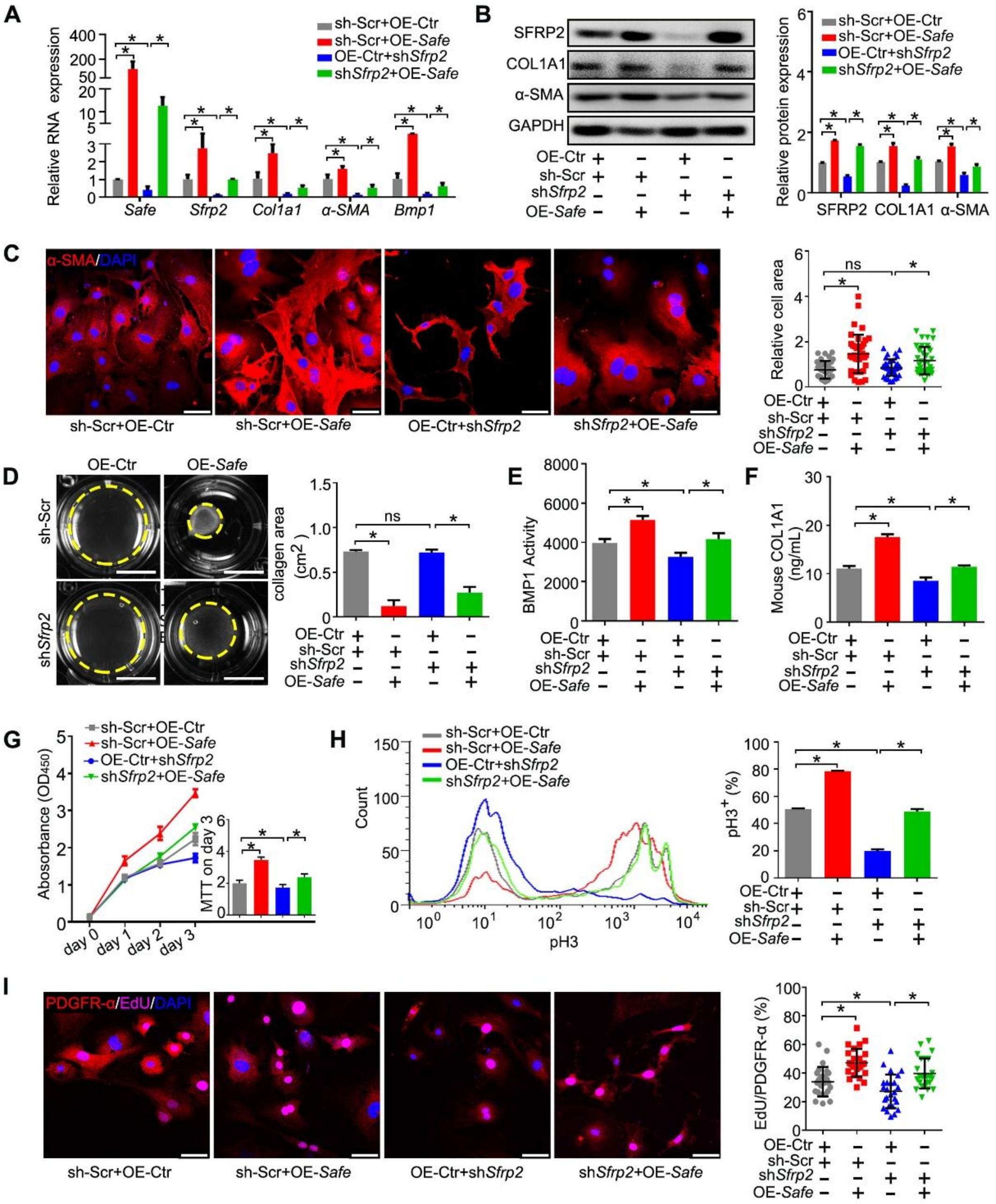

Figure S6 *Safe* overexpression disturbed the regulatory effects of sh*Sfrp2* in cardiac fibroblasts. (A) qRT-PCR analysis showing mRNA expression of *Safe*, *Sfrp2, Col1a1*, *α-SMA and Bmp1* by *Safe* overexpression in *Sfrp2*-silenced cardiac fibroblasts (n=3). (B) Representative western blot analysis and relative densitometric quantification showing up-regulated expression of SFRP2, COL1A1 and α-SMA proteins by *Safe* overexpression in *Sfrp2*-silenced cardiac fibroblasts (n=3). (C) Representative images of immunofluorescence staining for α-SMA (red) and quantification of the relative cell area of cardiac fibroblasts after treatments as indicated (n=40). Scale bar indicates 50 μm. (D) Representative images of collagen gel contraction for 24 hours and quantification of collagen area inside the dashed circles (n=3).

Scale bar indicates 0.5 cm. (E) BMP1 protein enzyme activity in the supernatant of cultured fibroblasts after indicated treatments (n=3). The excitation wavelength is 320 nm, while the emission wavelength is 405 nm. (F) ELISA assay of COL1A1 protein in the supernatant of cultured fibroblasts after indicated treatments (n=3). (G) CCK-8 assay of cardiac fibroblasts showing restored cell proliferation by *Safe* overexpression in *Sfrp2*-deficient cardiac fibroblasts (n=3). (H) Flow cytometry analysis of pH3 incorporation in *Safe* overexpressed-stimulated fibroblasts with indicated treatments. (I) Representative images of immunofluorescence staining for EdU (magenta), PDGFR-α (red) and DAPI (blue), n=4. Scale bar indicates 50 μm. Right panel: Percent of EdU^+ cells in PDGFR-α^+ cells, (n=25). All data are presented as mean ± SEM; Student's *t*-test or one-way ANOVA; *$p < 0.05$.

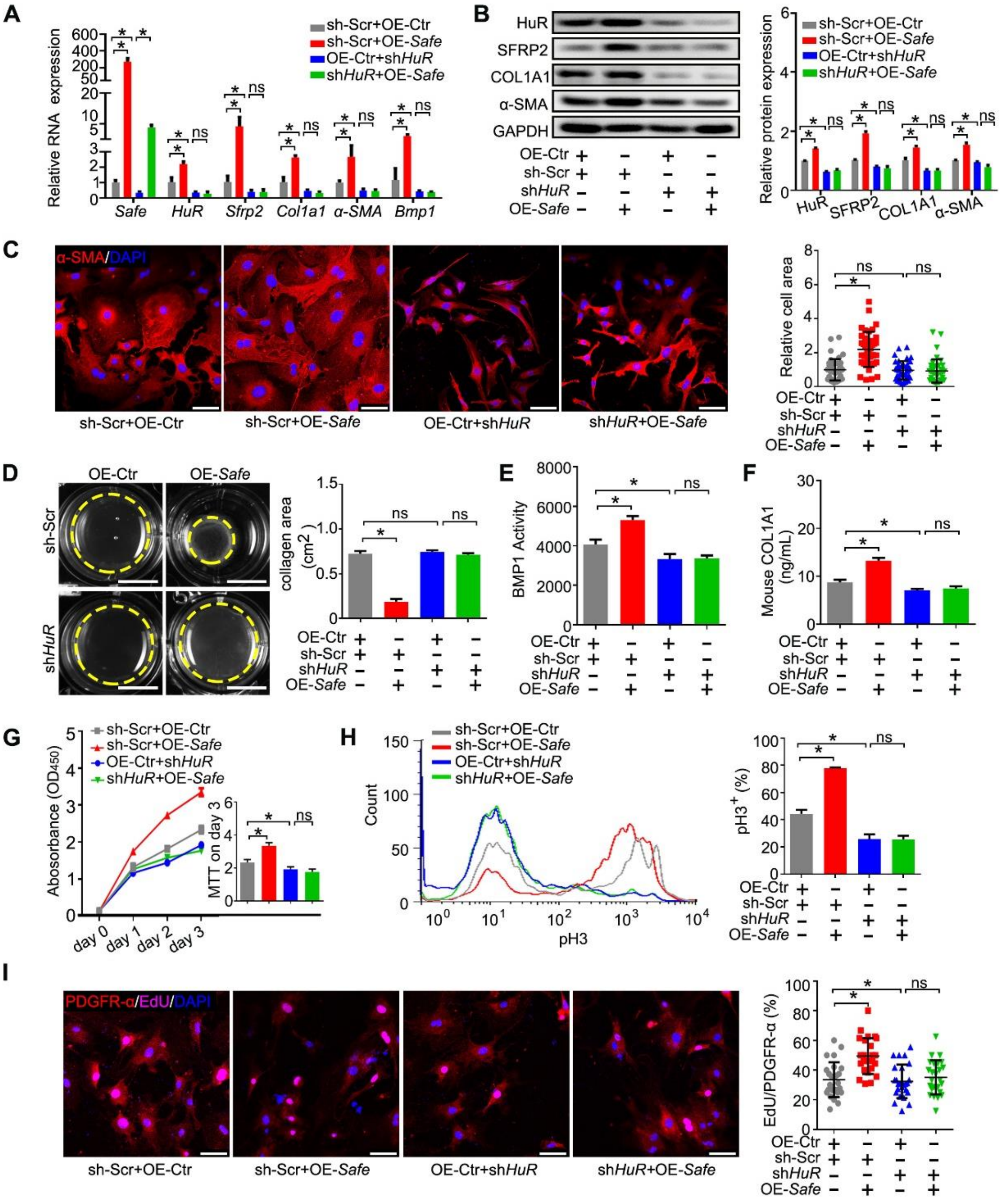

Figure S7 *Safe* overexpression failed to reverse the phenotype changes of cardiac fibroblasts induced by *HuR* shRNAs. (A) qRT-PCR analysis showing mRNA expression of *Safe*, *HuR*, *Sfrp2*, *Col1a1*, *α-SMA and Bmp1* by *Safe* overexpression in *HuR*-silenced cardiac fibroblasts (n=3). (B) Representative western blot analysis and relative densitometric quantification showing expression of HuR, SFRP2, COL1A1 and α-SMA proteins by *Safe* overexpression in *HuR*-silenced cardiac fibroblasts (n=3). (C) Representative images of immunofluorescence staining for α-SMA (red, n=5) and quantification of the relative cell area of cardiac fibroblasts after treatments as indicated (n=40). Scale bar indicates 50 μm. (D) Representative images of collagen gel contraction for 24 hours and quantification of collagen area

inside the dashed circles (n=3). Scale bar indicates 0.5 cm. (E) BMP1 protein enzyme activity in the supernatant of cultured fibroblasts after indicated treatments (n=3). The excitation wavelength is 320 nm, and the emission wavelength is 405 nm. (F) ELISA assay of COL1A1 protein in the supernatant of cultured fibroblasts after indicated treatments (n=3). (G) CCK-8 assay of cardiac fibroblasts showing failed to restore cell proliferation by *Safe* overexpression in *HuR*-deficient cardiac fibroblasts (n=3). (H) Flow cytometry analysis of pH3 incorporation in *Safe* overexpressed-stimulated fibroblasts with indicated treatments. (I) Representative images of immunofluorescence staining for EdU (magenta), PDGFR-α (red) and DAPI (blue). Scale bar indicates 50 μm. Right panel: Percent of EdU^+ cells in PDGFR-α^+ cells (n=25). All data are presented as mean ± SEM; Student's *t*-test or one-way ANOVA; *$p < 0.05$.

Table S1 The primer list for RACE assay, *in vitro* translation assay and construction of luciferase reporter pGL3-control vectors carrying various *Safe* fragments or the 3'-UTR of *Sfrp2*

Safe	5' RACE	GCCACTGTCAGCTCGGCTCCAACGC
	3' RACE	TCTGGCTGAAATGTGGGGTACATGCT
	1bp-1517 bp	Sense: GCTCTAGAAGAGCCCAGCAAGTAACCCTT
		Antisense: CCCCGGCCGGCCCCCTCTGTGGTCTAAGCTTGTGG
	1bp-528bp	Sense: GCTCTAGAAGAGCCCAGCAAGTAACCCTT
		Antisense: CCCCGGCCGGCCGAGGTACCACTGCAGCCACA
	529bp-1055bp	Sense: GCTCTAGACTCCGGAGCCCAGGTCAC
		Antisense: CCCCGGCCGGCCTTCTTTGTCTTCTGTCATGTCTCCT
	1056bp-1517bp	Sense: GCTCTAGATAGGTACAACAAGATGTAAAGTTTATATACAA
		Antisense: CCCCGGCCGGCCCCCTCTGTGGTCTAAGCTTGTGG
	938bp-1412bp	Sense: CCACTGGAGTAGCTGGTAACTTC
		Antisense: CAAACATTTCGTTGCTCGTTTT
	Sense-T7	Sense: TAATACGACTCACTATAAGAGCCCAGCAAGTAACCCTTTA
		Antisense: TTACCCTCTGTGGTCTAAGCTTGTGG
	Antisense-T7	Sense: TAATACGACTCACTATACCCTCTGTGGTCTAAGCTTGTG
		Antisense: TTAAGAGCCCAGCAAGTAACCCT
Sfrp2 (3'-UTR)	1110bp-2001bp	Sense: GCTCTAGA TTTCCCAGTGGGGTGGCTTC
		Antisense: CCCCGGCCGGCCTAGGTACAACAAGATGTAAAGTTTATATAC
	1110bp-1539bp	Sense: GCTCTAGACCCAGTGGGGTGGCTTCTCT
		Antisense: CCCCGGCCGGCCGCCTGACATGGCAACCTAAGTG
	1540bp-2001bp	Sense: GCTCTAGACCCTCTGTGGTCTAAGCTTGTGG
		Antisense: CCCCGGCCGGCCTGTTTATCTGAAATATTTACAGTGTCGG

Table S2 The sequences of shRNA or gRNA targeting *Safe*, *Sfrp2* and *HuR*, and primers used to amplify the full length of *Sfrp2* CDS region.

Safe	shRNA	Sense:<br>GATCCGAATCTCCAGCAATACACATTCAAGAGATGTGTATTGCTGGAGATTCTTTTTG<br>Antisense:<br>AATTCAAAAAGAATCTCCAGCAATACACATCTCTTGAATGTGTATTGCTGGAGATTCG
	gRNA1	Sense:<br>CACCGCATAGGTTTGAATCCGAACC<br>Antisense:<br>AAACGGTTCGGATTCAAACCTATGC
	gRNA2	Sense:<br>CACCGCGCGGGTACCTGCTCGAGCT<br>Antisense:<br>AAACAGCTCGAGCAGGTACCCGCGC
	Over-expression	Sense:<br>GCTCTAGAAGAGCCCAGCAAGTAACCCTT
		Antisense:<br>CGGAATTCCCCTCTGTGGTCTAAGCTTGTGG
Sfrp2	shRNA	Sense:<br>GATCCGCCCATCCAGATCTTGTACTTCAAGAGAGTACAAGATCTGGATGGGCTTTTTG<br>Antisense:<br>AATTCAAAAAGCCCATCCAGATCTTGTACTCTCTTGAAGTACAAGATCTGGATGGGCG
	Over-expression	Sense:<br>CGGAATTCGCCACCATGCCGCGGGGCCCTGCC<br>Antisense:<br>CGGGATCCCTAGCATTGCAGCTTGCGGATGC
HuR	shRNA	Sense:<br>GATCCGAAGCTGCAATGGCCATAGTTCAAGAGACTATGGCCATTGCAGCTTCTTTTTG<br>Antisense:<br>AATTCAAAAAGAAGCTGCAATGGCCATAGTCTCTTGAACTATGGCCATTGCAGCTTCG

Table S3 The primer list for qRT-PCR.

Gene name	Sense (5’→3’)	Antisense (5’→3’)
Safe	GGGAGAGTGTAGCAGAGTCC	ATGTAGGAGGACCCGCAGAA
Meg3	ACACACGGACACAGACACC	TGGGTCTCTCTACTCAAGGG
Sfrp2	CCACAGAGGAAGCTCCCAA	GGTGTCTCTGTTGATGTACGTT
AK045784	CTTCCTGTCCTTCCAAATAA	CTCGTGGCTTACTGCTATTC
AK039829	GACAAGAGGTTCCGCAGGGTA	CCATTCATTCCAGTTAGTCATCG
AK009088	TAGCCAATAAGCACAACAGC	GGTAGGTTGACTCTTCGGAA
AK009418	ACTTAGTTTCTGAGCCCTGA	ATTCCGTCCTGTAACAAAGA
uc008hbv	GCGTGCTCTATGAAGTACCA	TCTTCAGAGTTGTAGGCAGC
AK020603	GCTCTTGCTCCCTCTTATGCT	AGGCTCAATGACGATGCTTAGA
18S	GTAACCCGTTGAACCCCATT	CCATCCAATCGGTAGTAGCG
Gapdh	GGTGAAGGTCGGTGTGAACG	CTCGCTCCTGGAAGATGGTG
U6	GTGCTCGCTTCGGCAGCA	GGAACGCTTCACGAATTTGC
α-SMA	CCCCCTCTAGTGGTCAGGAA	ACGCTCTCAAATACCCCGTT
Col1a1	CCGAACCCCAAGGAAAAGA	GTGGACATTAGGCGCAGGA
FSp1	GCTGCCCAGATAAGGAACCC	TGCGAAGAAGCCAGAGTAAGG
Myh7	AAGGGCCTGAATGAGGAGTA	AAAGGCTCCAGGTCTGAGG
HuR	AAACATGGCTCTCCTCTCGC	ACCCATAGGGGAGAACCTGA
Pecam1	GGCACACCTGTAGCCAACTTC	GCCGCTTCTCTTGACCACTT
Bmp1	ACTACACCTACGACCTGGGC	TTTGATGGATGGCCTTCGGG
Postn	TGTGGGGTAGGAACTGAGGA	CTTCCTGAGGCGGAAGGTTT
Col3a1	TCCCCTGGAATCTGTGAATC	TGAGTCGAATTGGGGAGAAT

Table S4 The probe list used in EMSA.

Probe 1F-biotin	AAGUUUAUAUACAAAGAAUAUAAUGUUUAU
Probe 1R-unlabeled	AUAAACAUUAUAUUCUUUGUAUAUAAACUU
Probe 2F-biotin	CUACAAUGAUUUAGUUUGCAAAAAAAAUGA
Probe 2R-unlabeled	UCAUUUUUUUUGCAAACUAAAUCAUUGUAG
Probe 3F-biotin	UGAAUUAAAAGAUGUUUUAAACAAAGCUUA
Probe 3R-unlabeled	UAAGCUUUGUUUAAAACAUCUUUUAAUUCA
Probe 4F-biotin	CGAGCAACGAAAUGUUUGAAGACUUAGAUG
Probe-4R-unlabeled	CAUCUAAGUCUUCAAACAUUUCGUUGCUCG

Table S5 The lncRNAs differentially expressed between tissues from infarct zone (IZ) and remote zone (RZ).

ProbeName	IZ-1	IZ-2	RZ-1	RZ-2	p_IZ_vs_RZ	fold_IZ_vs_RZ	GeneSymbol	SeqID	location	overlapped_gene
CUST_5502_PI419771968	14.84883	14.639	10.29681	9.745277	0.019575	4.722875	Ptn	uc009biz	Intergenic	NA
CUST_13004_PI419771968	10.19638	8.878916	4.003442	5.630894	0.049556	4.720479	NA	AK169965	Sense	Mmp12
CUST_12561_PI419771968	9.72405	8.946613	4.140127	5.14823	0.021215	4.691153	NA	AK089297	Sense	Gpnmb
CUST_12946_PI419771968	9.149141	9.814359	5.580841	4.917067	0.0121	4.232796	NA	AK162395	Sense	Kcnma1
CUST_3617_PI419771968	8.451489	9.124411	5.290115	4.304183	0.029488	3.990801	AK077272	uc008afh	Intergenic	NA
CUST_10732_PI419771968	9.605808	9.325877	5.568197	5.583089	0.022525	3.8902	NA	AK044494	Sense	Klhl29
CUST_2235_PI419771968	10.50582	10.43204	6.802367	6.378015	0.030033	3.878736	AK136017	uc007ile	Sense	Slit3
CUST_12083_PI419771968	9.715947	10.31435	6.740719	5.697182	0.04096	3.796197	NA	AK082557	Antisense	Gm11149
CUST_1510_PI419771968	16.76071	16.36178	12.95679	12.59084	0.00522	3.787429	NA	AK159300	Sense	Col1a1
CUST_10889_PI419771968	9.812981	8.71857	5.073944	6.12672	0.040452	3.665444	NA	AK046559	Sense	Ms4a7
CUST_781_PI419771968	13.58794	13.62626	10.26196	9.836924	0.036548	3.557659	NA	AK046686	Intergenic	NA
CUST_8583_PI419771968	10.17487	9.915772	6.746323	6.261502	0.015301	3.541406	NA	AK018031	Sense	Ptk2b
CUST_2689_PI419771968	7.279145	7.820086	4.327397	4.051961	0.020911	3.359937	Gtl2	uc007pat	Intergenic	NA
CUST_10588_PI419771968	8.573981	8.133397	5.228601	4.774037	0.008827	3.352371	NA	AK042906	Sense	Unc5c
CUST_2687_PI419771968	13.67454	13.16217	10.25275	9.90526	0.013017	3.339349	Gtl2	uc007par	Intergenic	NA
CUST_10145_PI419771968	10.44971	10.00365	6.800575	7.053255	0.013706	3.299763	NA	AK037708	Sense	Runx1
CUST_13039_PI419771968	8.540566	8.912185	5.402096	5.470919	0.030476	3.289868	NA	AB063319	Intergenic	NA
CUST_10257_PI419771968	10.33183	10.57825	7.117573	7.215513	0.010416	3.288496	NA	AK038732	Sense	Gria3
CUST_8766_PI419771968	7.608441	7.324596	4.387299	4.059848	0.004812	3.242945	NA	AK019526	Sense	Loxl1
CUST_11344_PI419771968	11.36858	11.55971	8.506542	8.027197	0.025639	3.197275	NA	AK051693	Intergenic	NA
CUST_1029_PI419771968	11.57126	11.09548	8.350982	7.95395	0.010396	3.180903	NA	AK081334	Intergenic	NA
CUST_12423_PI419771968	6.040756	6.463502	3.465196	2.767417	0.027746	3.135823	NA	AK086575	Sense	Dclre1c
CUST_9899_PI419771968	12.04279	11.81491	8.847862	8.750848	0.009	3.129492	NA	AK035258	Sense	Synpo2
CUST_5413_PI419771968	10.45513	10.16648	7.569342	6.862988	0.04332	3.09464	AK008014	uc008zxj	Antisense	Abhd11
CUST_11987_PI419771968	9.846146	9.361053	6.292346	6.742613	0.011505	3.08612	NA	AK081565	Intergenic	NA
CUST_12722_PI419771968	9.612109	9.312686	6.569526	6.236202	0.005561	3.059534	NA	AK137012	Sense	Csmd1
CUST_2690_PI419771968	11.62414	11.5817	8.803762	8.351896	0.045695	3.025087	Gtl2	uc007pav	Intergenic	NA
CUST_9920_PI419771968	8.386811	8.345634	5.308845	5.373652	0.000509	3.024974	NA	AK035449	Sense	Foxp2

CUST_4263_PI419771968	7.165568	7.658231	4.322906	4.481532	0.034763	3.009681	AK138201	uc008jko	Antisense	Dab2ip
CUST_2695_PI419771968	11.58511	11.76209	8.934245	8.411436	0.03607	3.000761	AJ517767	uc007pbd	Intergenic	NA
CUST_9717_PI419771968	10.52472	10.79638	8.029128	7.42673	0.034219	2.93262	NA	AK033438	Sense	Lmo4
CUST_2685_PI419771968	13.46428	12.81267	9.997694	10.55047	0.023022	2.864398	AK157178	uc007pap	Intergenic	NA
CUST_683_PI419771968	7.89675	8.011162	5.134735	5.080219	0.003148	2.846479	NA	AK039829	Intergenic	NA
CUST_10842_PI419771968	8.957013	9.043117	6.340978	5.976472	0.031749	2.84134	NA	AK045784	Intergenic	NA
CUST_11897_PI419771968	8.393419	8.847528	6.108392	5.512974	0.020801	2.80979	NA	AK080487	Sense	Unc5c
CUST_11141_PI419771968	8.515246	8.002893	5.218044	5.689126	0.01535	2.805485	NA	AK048773	Sense	Kcnma1
CUST_4671_PI419771968	8.978911	8.538985	5.558345	6.379756	0.048344	2.789898	AK137033	uc008ppm	Antisense	Sfrp2
CUST_3208_PI419771968	7.062229	7.570456	4.621547	4.471764	0.042223	2.769687	BC023070	uc007vda	Intergenic	NA
CUST_1895_PI419771968	10.81545	10.4075	8.199606	7.50637	0.034728	2.758485	AK145301	uc007edt	Antisense	Sertad4
CUST_10583_PI419771968	9.901871	9.776586	7.263694	6.90619	0.024386	2.754287	NA	AK042874	Sense	Lrrc16a
CUST_10933_PI419771968	9.049584	8.726994	6.089533	6.181569	0.025626	2.752738	NA	AK046963	Sense	Cdk14
CUST_9645_PI419771968	10.49411	10.90232	8.046375	7.848131	0.019813	2.750963	NA	AK032779	Sense	Ncam1
CUST_2448_PI419771968	10.02906	9.901371	7.332767	7.130272	0.004225	2.733697	Gm885	uc007lzk	Antisense	Polg2
CUST_7718_PI419771968	12.10429	12.17048	9.474507	9.455208	0.004108	2.672525	NA	AK014707	Sense	Enpp1
CUST_10908_PI419771968	9.673073	10.36822	7.784429	6.964737	0.040954	2.646063	NA	AK046743	Sense	Dclk1
CUST_11755_PI419771968	7.891607	7.927107	5.189428	5.342434	0.013284	2.643426	NA	AK078197	Sense	Runx2
CUST_7403_PI419771968	7.266341	6.983739	4.721337	4.314011	0.013068	2.607366	NA	AK011385	Sense	Nav1
CUST_1010_PI419771968	10.62572	10.25362	7.916143	7.76247	0.023179	2.600363	NA	AK080501	Intergenic	NA
CUST_12897_PI419771968	9.629893	9.574684	6.981317	7.024742	0.000268	2.599259	NA	AK155750	Sense	Lilrb4
CUST_12975_PI419771968	7.798883	7.563833	5.106144	5.140759	0.026095	2.557907	NA	AK164022	Sense	Itga4
CUST_12563_PI419771968	8.630685	8.676999	6.161964	6.063295	0.003104	2.541213	NA	AK089314	Sense	Lilrb4
CUST_6342_PI419771968	8.430329	8.02405	5.372071	6.002295	0.031182	2.540007	AK004221	uc009nrm	Antisense	Gm20388
CUST_11357_PI419771968	10.02194	9.740517	7.564173	7.136129	0.015998	2.531078	NA	AK051822	Sense	Tgfb2
CUST_10872_PI419771968	10.17979	10.37406	7.943686	7.559042	0.019489	2.525559	NA	AK046232	Sense	Serpine2
CUST_9065_PI419771968	8.763352	8.35335	5.867248	6.21923	0.012189	2.515112	NA	AK020555	Sense	Antxr1
CUST_3165_PI419771968	8.497348	8.593107	5.972018	6.154403	0.006156	2.482017	AK079958	uc007uwp	Intergenic	NA
CUST_8364_PI419771968	9.589382	9.135541	7.082455	6.695372	0.015299	2.473548	NA	AK017175	Sense	Adam12
CUST_9271_PI419771968	8.232893	8.242259	5.791991	5.778211	3.87E-05	2.452475	NA	AK021285	Intergenic	NA
CUST_10899_PI419771968	8.000402	8.283775	5.985748	5.406669	0.038355	2.44588	NA	AK046626	Antisense	Bnc2

CUST_9094_PI419771968	12.66715	12.75244	10.49048	10.09285	0.043405	2.41813	NA	AK020657	Sense	Pdzrn3
CUST_9458_PI419771968	10.29846	9.923447	7.712616	7.68425	0.048202	2.412521	NA	AK030568	Intergenic	NA
CUST_11010_PI419771968	8.913722	8.191298	5.946702	6.367089	0.046681	2.395615	NA	AK047681	Sense	Pcdh9
CUST_8952_PI419771968	10.81215	10.58968	8.36908	8.356112	0.029731	2.338314	NA	AK020229	Sense	Stambpl1
CUST_8380_PI419771968	10.45087	9.858559	7.493247	8.146379	0.034539	2.3349	NA	AK017233	Sense	Aff3
CUST_1572_PI419771968	11.30132	10.9338	8.855254	8.711308	0.027841	2.334278	NA	AK168001	Intergenic	NA
CUST_7538_PI419771968	7.001927	6.701077	4.504325	4.539449	0.03857	2.329615	NA	AK013470	Intergenic	NA
CUST_12801_PI419771968	8.231805	8.115468	6.036429	5.651734	0.03672	2.329555	NA	AK142062	Sense	Ptprj
CUST_8702_PI419771968	9.670571	8.962562	6.737577	7.270058	0.040571	2.312749	NA	AK018648	Sense	Itpk1
CUST_4459_PI419771968	6.515592	6.910253	4.561649	4.274797	0.014768	2.2947	AK031463	uc008mrt	Intergenic	NA
CUST_11731_PI419771968	11.15449	10.99798	8.936894	8.634321	0.015246	2.290626	NA	AK077892	Sense	Kalrn
CUST_9754_PI419771968	11.5403	11.25984	9.196724	9.034686	0.011401	2.284367	NA	AK034008	Sense	Pcdh9
CUST_6440_PI419771968	13.97512	13.84392	11.67676	11.58262	0.002077	2.27983	AK141402	uc009pal	Sense	2610203C20Rik
CUST_2097_PI419771968	10.75548	10.42422	8.479392	8.249958	0.012029	2.225177	AK038827	uc007gvs	Sense	Tmcc3
CUST_9012_PI419771968	8.5511	8.21229	6.320454	6.032687	0.010895	2.205125	NA	AK020424	Sense	Odz3
CUST_1780_PI419771968	9.967793	10.30537	8.098991	7.782277	0.01107	2.195948	AK192563	uc007ctb	Sense	Shisa4
CUST_11233_PI419771968	8.172631	8.457986	6.332979	5.910809	0.019356	2.193415	NA	AK050122	Sense	Apol9a
CUST_3190_PI419771968	9.715022	9.251226	7.17997	7.415363	0.031492	2.185458	B930095G15Rik	uc007vab	Sense	Farp1
CUST_9398_PI419771968	12.94391	12.65599	10.61772	10.61549	0.041903	2.183347	NA	AK029601	Sense	Bicc1
CUST_940_PI419771968	9.684195	9.745506	7.633944	7.439393	0.017148	2.178182	NA	AK078071	Sense	Celf4
CUST_9276_PI419771968	10.17518	9.964663	7.666795	8.173914	0.044621	2.149565	NA	AK021303	Sense	Ccdc80
CUST_12998_PI419771968	12.16504	12.10751	10.16419	9.822019	0.045138	2.143171	NA	AK166199	Intergenic	NA
CUST_10074_PI419771968	11.28097	11.34915	9.337783	9.082014	0.027948	2.105161	NA	AK037075	Sense	Arid5b
CUST_10249_PI419771968	7.499063	7.394799	5.42523	5.284966	0.002574	2.091833	NA	AK038647	Intergenic	NA
CUST_9837_PI419771968	8.765991	9.124508	7.099331	6.620612	0.024301	2.085278	NA	AK034700	Sense	Rai14
CUST_9266_PI419771968	8.108993	8.532529	6.32752	6.149996	0.036367	2.082003	NA	AK021264	Sense	Nfat5
CUST_6906_PI419771968	8.798566	8.487	6.559456	6.636988	0.037486	2.044561	NA	AK005401	Intergenic	NA
CUST_8528_PI419771968	9.312966	9.112044	7.096156	7.259057	0.004778	2.034899	NA	BC028775	Sense	Adc
CUST_9340_PI419771968	13.09967	12.93693	11.13606	10.84748	0.014976	2.026532	NA	AK028812	Sense	Adamts12
CUST_13098_PI419771968	12.0656	11.87417	10.03094	9.877609	0.004484	2.015612	NA	AY039009	Sense	Zeb2
CUST_12355_PI419771968	12.24533	11.8896	10.22141	9.884604	0.014592	2.014457	NA	AK085840	Sense	Lhfp

CUST_10810_PI419771968	8.232433	8.2445	6.296862	6.161906	0.020391	2.009083	NA	AK045346	Sense	Plod2
CUST_1601_PI419771968	8.421913	8.735368	6.706991	6.45385	0.011537	1.99822	NA	AK172317	Sense	4933407C03Rik
CUST_3813_PI419771968	8.417148	8.526662	6.672319	6.31878	0.039704	1.976356	AK006637	uc008dqd	Antisense	Cyp1b1
CUST_10182_PI419771968	10.88409	10.72819	9.038426	8.622428	0.042031	1.975713	NA	AK038070	Sense	Hivep3
CUST_9260_PI419771968	7.197065	7.161802	5.073472	5.338131	0.039357	1.973632	NA	AK021240	Sense	Greb1l
CUST_11631_PI419771968	10.47987	10.38742	8.472204	8.472233	0.015001	1.961426	NA	AK054342	Sense	Lhfpl2
CUST_12678_PI419771968	11.43352	11.7985	9.631416	9.726647	0.046488	1.936977	NA	AK133634	Sense	Tram2
CUST_9149_PI419771968	7.251189	7.548305	5.628564	5.328649	0.011861	1.921141	NA	AK020866	Intergenic	NA
CUST_12920_PI419771968	12.00903	11.77669	10.06044	9.901515	0.008561	1.911881	NA	AK158356	Sense	Zeb2
CUST_8903_PI419771968	7.610513	7.862996	6.091981	5.565556	0.048554	1.907986	NA	AK020075	Sense	Grk5
CUST_10102_PI419771968	8.349133	8.089117	6.283845	6.36089	0.02994	1.896758	NA	AK037318	Sense	Eif4e3
CUST_1379_PI419771968	15.204	15.32036	13.37397	13.37444	0.01961	1.887976	NA	AK149239	Sense	Dpysl3
CUST_1016_PI419771968	10.39557	9.973862	8.054598	8.540878	0.029185	1.886977	NA	AK080649	Intergenic	NA
CUST_9110_PI419771968	10.71476	10.57812	8.831577	8.687364	0.002797	1.886968	NA	AK020712	Sense	Spats2l
CUST_12241_PI419771968	10.76123	10.58119	8.809562	8.759673	0.020649	1.886593	NA	AK084442	Sense	Foxp1
CUST_587_PI419771968	13.32023	13.32467	11.53677	11.34101	0.032967	1.883558	NA	AK031998	Sense	Scrn1
CUST_13475_PI419771968	11.25575	11.30154	9.394946	9.423522	0.000676	1.869411	H19	NR_001592	Intergenic	NA
CUST_5516_PI419771968	9.164212	8.808272	6.940833	7.309498	0.018494	1.861077	AK047163	uc009bma	Antisense	Dennd2a
CUST_8904_PI419771968	9.179685	9.298352	7.287408	7.478613	0.007569	1.856008	NA	AK020076	Sense	Nrip1
CUST_12374_PI419771968	8.237918	8.452827	6.663204	6.316588	0.020313	1.855477	NA	AK086137	Sense	4933407C03Rik
CUST_8886_PI419771968	10.81276	10.40314	8.648559	8.876313	0.030387	1.845514	NA	AK019982	Sense	Tanc2
CUST_10424_PI419771968	8.265396	8.385642	6.694033	6.310753	0.047666	1.823126	NA	AK040785	Antisense	Ly96
CUST_9902_PI419771968	8.590548	8.265377	6.573745	6.714886	0.030296	1.783647	NA	AK035284	Sense	Foxp1
CUST_6800_PI419771968	11.84734	11.73158	10.01583	10.0264	0.019824	1.768342	Sat1	uc009urr	Sense	Sat1
CUST_8908_PI419771968	7.830511	7.498735	5.7447	6.070334	0.01707	1.757106	NA	AK020094	Sense	Plekhg3
CUST_11569_PI419771968	8.771661	8.58195	6.957395	6.908689	0.025235	1.743764	NA	AK053547	Sense	Map2k6
CUST_8923_PI419771968	7.317548	7.200697	5.581557	5.453367	0.002571	1.741661	NA	AK020138	Sense	6720463M24Rik
CUST_12196_PI419771968	7.912072	7.736775	6.142276	6.023972	0.006287	1.7413	NA	AK083891	Sense	Ern1
CUST_2210_PI419771968	6.186753	6.530843	4.697191	4.581876	0.043006	1.719265	AK087392	uc007ief	Intergenic	NA
CUST_13536_PI419771968	13.21399	13.1725	11.59648	11.40104	0.029642	1.694487	Pisd-ps1	NR_003517	Antisense	Sfi1
CUST_10690_PI419771968	8.354147	8.287149	6.62137	6.667021	0.001217	1.676453	NA	AK044029	Antisense	Chadl

CUST_9328_PI419771968	10.10897	10.26736	8.705442	8.331214	0.040975	1.669834	NA	AK028519	Sense	10-Sep
CUST_11970_PI419771968	6.243261	6.708535	4.986406	4.626539	0.034004	1.669426	NA	AK081389	Intergenic	NA
CUST_6439_PI419771968	11.44489	10.95048	9.277193	9.781371	0.042014	1.668405	AK011865	uc009pak	Sense	2610203C20Rik
CUST_5168_PI419771968	7.598713	7.545215	6.034674	5.77683	0.041124	1.666212	AK012891	uc008wse	Antisense	Smarcd3
CUST_10343_PI419771968	8.202883	8.328236	6.44802	6.768742	0.036402	1.657179	NA	AK039696	Sense	St6gal1
CUST_11400_PI419771968	8.249525	8.343602	6.738688	6.546036	0.013861	1.654202	NA	AK052106	Sense	Pbx1
CUST_7503_PI419771968	8.842567	9.012122	7.331146	7.255072	0.013425	1.634236	NA	AK013025	Sense	Fam115a
CUST_8388_PI419771968	11.30277	11.25237	9.779413	9.519427	0.043482	1.628149	NA	AK017285	Sense	Clcn5
CUST_12297_PI419771968	11.14113	10.97488	9.494727	9.374943	0.005854	1.623166	NA	AK085124	Sense	Myo1d
CUST_4531_PI419771968	7.953968	8.12483	6.299384	6.540267	0.011676	1.619574	AK077405	uc008nzz	Antisense	Ube2v1
CUST_3535_PI419771968	8.835628	8.465323	6.940088	7.155253	0.030816	1.602806	AK028852	uc007znc	Sense	Tbc1d23
CUST_7625_PI419771968	8.605184	8.558467	7.002679	6.958291	0.000412	1.601341	NA	AK013908	Intergenic	NA
CUST_4893_PI419771968	8.461783	8.587887	7.040975	6.84905	0.009089	1.579823	AK172190	uc008sru	Sense	Rnf38
CUST_2281_PI419771968	13.21568	13.19948	11.76377	11.5199	0.048563	1.565745	AK157519	uc007jjl	Intergenic	NA
CUST_9479_PI419771968	13.58468	13.36624	11.8064	12.02406	0.009626	1.56023	NA	AK031010	Antisense	4933403F05Rik
CUST_2056_PI419771968	13.91145	13.68442	12.26835	12.23675	0.042945	1.545384	AK200837	uc007ghs	Sense	F630110N24Rik
CUST_11579_PI419771968	6.548323	6.556582	4.956793	5.060677	0.020631	1.543718	NA	AK053637	Intergenic	NA
CUST_8669_PI419771968	9.141201	9.06424	7.578083	7.559961	0.011229	1.533698	NA	AK018479	Sense	Bod1l
CUST_8450_PI419771968	13.9244	13.8613	12.40952	12.31057	0.003268	1.532803	NA	AK017619	Sense	Dab2
CUST_13696_PI419771968	6.616962	6.87782	5.069108	5.364958	0.016934	1.530358	LOC100270707	NR_026741	Intergenic	NA
CUST_12074_PI419771968	6.728229	6.884006	5.36325	5.197047	0.005615	1.525969	NA	AK082465	Intergenic	NA
CUST_11757_PI419771968	10.55673	10.47869	9.015701	8.990748	0.008734	1.514481	NA	AK078202	Sense	Fchsd2
CUST_9935_PI419771968	10.17689	9.942833	8.584631	8.535407	0.041303	1.499844	NA	AK035600	Sense	Calu
CUST_6636_PI419771968	7.887715	8.010697	6.537856	6.368151	0.006986	1.496203	AK086087	uc009rze	Antisense	Rbms3
CUST_9219_PI419771968	11.94697	11.72678	10.4022	10.3033	0.021968	1.484122	NA	AK021072	Sense	Myo1d
CUST_8466_PI419771968	7.018642	6.866139	5.445799	5.502995	0.017147	1.467993	NA	AK017752	Sense	Nr6a1
CUST_3_PI419771968	12.97807	12.856	11.4909	11.40826	0.004479	1.467456	NA	AK002304	Intergenic	NA
CUST_11805_PI419771968	9.195639	9.003843	7.658459	7.63928	0.040155	1.450872	NA	AK078845	Sense	Ube2e3
CUST_9720_PI419771968	11.7336	11.94127	10.57813	10.23107	0.032211	1.43284	NA	AK033584	Sense	Hivep2
CUST_2554_PI419771968	7.130738	6.781587	5.400654	5.690153	0.026986	1.410759	AK133650	uc007ndz	Intergenic	NA
CUST_11927_PI419771968	10.06263	10.10218	8.774616	8.593546	0.033229	1.398327	NA	AK080924	Sense	Snx10

CUST_12717_PI419771968	8.738371	8.653303	7.3278	7.269641	0.002538	1.397117	NA	AK136865	Sense	1110003E01Rik
CUST_10207_PI419771968	10.1413	9.893524	8.756625	8.499561	0.016175	1.389317	NA	AK038224	Intergenic	NA
CUST_10576_PI419771968	8.527037	8.643803	7.248537	7.15019	0.003428	1.386057	NA	AK042823	Sense	Zfpm2
CUST_13487_PI419771968	15.61774	15.67082	14.30887	14.21673	0.004334	1.381483	Gas5	NR_002840	Intergenic	NA
CUST_11282_PI419771968	6.674732	6.80452	5.50151	5.21977	0.033205	1.378986	NA	AK051030	Sense	Tax1bp1
CUST_12631_PI419771968	7.227922	7.50187	6.200716	5.775181	0.044868	1.376948	NA	AK090243	Sense	Lman2l
CUST_4974_PI419771968	9.810852	9.826187	8.459043	8.432375	0.000608	1.372811	2210012G02Rik	uc008tzi	Sense	2210012G02Rik
CUST_2317_PI419771968	7.652344	7.415448	5.986886	6.372523	0.040745	1.354192	AK039862	uc007kcg	Sense	Pafah1b1
CUST_9937_PI419771968	10.30595	10.6829	8.95985	9.325106	0.035752	1.351949	NA	AK035610	Intergenic	NA
CUST_1584_PI419771968	5.889178	5.735608	4.502136	4.430245	0.014367	1.346203	NA	AK170083	Sense	Acox3
CUST_6297_PI419771968	9.812264	9.687542	8.53255	8.280045	0.027244	1.343606	AK080383	uc009ndl	Antisense	Ctcf
CUST_6869_PI419771968	10.33394	10.44377	9.186918	8.915192	0.037568	1.337797	NA	AK003925	Intergenic	NA
CUST_2362_PI419771968	8.749777	8.533183	7.515946	7.121486	0.048218	1.322764	AK011568	uc007kry	Antisense	Bcas3
CUST_3727_PI419771968	7.662371	7.426777	6.264238	6.188719	0.038744	1.318096	AK140218	uc008cad	Antisense	Zbtb22
CUST_11029_PI419771968	10.04718	9.984481	8.77175	8.632165	0.01385	1.313871	NA	AK047823	Sense	Sap30bp
CUST_12069_PI419771968	9.353048	9.199527	7.844719	8.08392	0.018709	1.311968	NA	AK082418	Sense	Mast4
CUST_3387_PI419771968	6.247055	6.189544	5.003447	4.837125	0.02431	1.298014	AK163711	uc007xjz	Intergenic	NA
CUST_7575_PI419771968	9.964509	10.06368	8.83798	8.604056	0.03075	1.293079	NA	AK013635	Sense	Prkg1
CUST_4431_PI419771968	8.385321	8.063993	7.103521	6.78962	0.029558	1.278086	AK157218	uc008mgp	Intergenic	NA
CUST_12519_PI419771968	8.878784	8.739309	7.56807	7.498512	0.012222	1.275756	NA	AK087847	Sense	Srp54c
CUST_8625_PI419771968	8.716217	8.669227	7.333195	7.507724	0.031896	1.272263	NA	AK018273	Sense	Ubxn2b
CUST_11428_PI419771968	11.17547	11.08586	9.937287	9.787828	0.010032	1.268105	NA	AK052455	Sense	Ddi2
CUST_7719_PI419771968	9.71067	10.04906	8.795788	8.438527	0.036153	1.262706	NA	AK014710	Sense	Akap13
CUST_10085_PI419771968	11.32599	11.27408	10.0818	10.01476	0.001585	1.251755	NA	AK037153	Sense	Ypel2
CUST_8601_PI419771968	7.694602	7.516271	6.523434	6.2096	0.036408	1.23892	NA	AK018162	Sense	Cables1
CUST_10223_PI419771968	7.075175	6.876693	5.862849	5.612488	0.018718	1.238266	NA	AK038358	Antisense	Dhx30
CUST_9530_PI419771968	9.886454	9.939049	8.712882	8.654952	0.001065	1.228835	NA	AK031438	Sense	Cmtm4
CUST_6899_PI419771968	11.74644	11.63236	10.521	10.4148	0.004133	1.221501	NA	AK005234	Sense	Rbm39
CUST_9104_PI419771968	8.514076	8.426206	7.355299	7.14429	0.029511	1.220347	NA	AK020687	Sense	Rinl
CUST_8919_PI419771968	9.050163	9.162387	7.879545	7.894626	0.026638	1.21919	NA	AK020122	Sense	Nbea
CUST_2271_PI419771968	7.278092	7.361945	5.988542	6.234067	0.043195	1.208715	AK030920	uc007jeg	Antisense	Zkscan17

CUST_13587_PI419771968	8.050871	8.196784	6.910063	6.921089	0.037372	1.208252	Xist	NR_001570	Intergenic	NA
CUST_5504_PI419771968	14.80724	14.88252	13.65515	13.63556	0.013418	1.199521	Creb3l2	uc009bje	Sense	Creb3l2
CUST_13113_PI419771968	14.79781	14.83046	13.63366	13.62321	0.004101	1.185697	NA	BC006792	Sense	Creb3l2
CUST_3684_PI419771968	10.26769	10.14798	9.143744	8.901217	0.030823	1.185355	AK079380	uc008bbb	Intergenic	NA
CUST_5948_PI419771968	10.04396	10.13309	9.020841	8.787705	0.037949	1.18425	AK088822	uc009idh	Sense	Eftud1
CUST_5544_PI419771968	11.76169	11.78172	10.66404	10.51365	0.037253	1.182858	AK129160	uc009buo	Intergenic	NA
CUST_7666_PI419771968	10.47481	10.54812	9.372037	9.310297	0.001931	1.170297	NA	AK014176	Sense	Ano6
CUST_6296_PI419771968	9.348063	9.454502	8.35851	8.10766	0.038618	1.168198	AK086852	uc009ndk	Antisense	Ctcf
CUST_12073_PI419771968	6.379309	6.550046	5.468092	5.144308	0.045387	1.158478	NA	AK082454	Antisense	6530409C15Rik
CUST_12321_PI419771968	5.263349	5.395817	4.173855	4.176525	0.036415	1.154393	NA	AK085434	Sense	Efna5
CUST_4937_PI419771968	9.100419	9.002166	7.881438	7.934934	0.007266	1.143107	AK029637	uc008tld	Sense	4930473A06Rik
CUST_2709_PI419771968	11.4081	11.6174	10.53278	10.21222	0.037978	1.140255	V00821	uc007pgs	Antisense	Gm11029
CUST_4987_PI419771968	8.52519	8.757837	7.691102	7.322553	0.049323	1.134686	AK048089	uc008ued	Intergenic	NA
CUST_12736_PI419771968	6.99552	7.097289	5.998455	5.830631	0.014329	1.131862	NA	AK138072	Intergenic	NA
CUST_4498_PI419771968	11.77243	11.5795	10.57901	10.51001	0.033572	1.131453	Caper	uc008nmw	Sense	Rbm39
CUST_1536_PI419771968	11.79739	11.89648	10.85693	10.59177	0.048683	1.122579	NA	AK162901	Sense	Ar
CUST_8484_PI419771968	8.287104	7.970426	6.865303	7.1512	0.035146	1.120514	NA	AK017916	Sense	Fam188a
CUST_12163_PI419771968	8.640406	8.375385	7.265111	7.525168	0.026747	1.112756	NA	AK083553	Sense	Bmpr2
CUST_9657_PI419771968	9.916083	9.789511	8.878622	8.758232	0.007119	1.03437	NA	AK032935	Sense	Khdrbs1
CUST_10755_PI419771968	8.761527	8.979748	7.937462	7.748269	0.020221	1.027772	NA	AK044830	Sense	Pou2f1
CUST_6631_PI419771968	11.61239	11.43248	12.4603	12.58691	0.016476	-1.00117	AK180240	uc009rxz	Sense	Dync1li1
CUST_10338_PI419771968	7.767477	7.768236	8.824569	8.716514	0.034261	-1.00269	NA	AK039666	Sense	Pde4b
CUST_12363_PI419771968	5.457063	5.290321	6.396065	6.360232	0.043826	-1.00446	NA	AK085973	Intergenic	NA
CUST_1382_PI419771968	9.692492	9.580487	10.52348	10.75898	0.038918	-1.00474	NA	AK149390	Antisense	Hectd2
CUST_9437_PI419771968	4.994886	4.840533	6.041811	5.80377	0.028698	-1.00508	NA	AK030301	Intergenic	NA
CUST_6336_PI419771968	5.681895	5.406355	6.691013	6.457125	0.031214	-1.02994	Mlycd	uc009npo	Antisense	Mlycd
CUST_1557_PI419771968	7.978007	8.040539	8.97802	9.105215	0.014882	-1.03234	NA	AK165621	Sense	Tmem140
CUST_5927_PI419771968	5.11368	5.171466	6.219857	6.133041	0.004699	-1.03388	AK043101	uc009hqy	Antisense	Rgma
CUST_498_PI421760821	7.846767	7.70778	8.685801	8.939883	0.036037	-1.03557	NA	uc.18-	Sense	Eri3
CUST_4947_PI419771968	7.870762	7.732262	8.797808	8.882797	0.011823	-1.03879	AK080222	uc008trz	Intergenic	NA
CUST_9141_PI419771968	10.72567	10.39801	11.49306	11.73137	0.042469	-1.05038	NA	AK020839	Sense	Gm9955

CUST_4767_PI419771968	5.943375	5.972746	7.047977	6.972555	0.010131	-1.05221	Ptbp2	uc008rdm	Sense	Ptbp2
CUST_1969_PI419771968	5.444157	5.312572	6.467344	6.44741	0.034834	-1.07901	AK006037	uc007eyj	Antisense	Armc2
CUST_7557_PI419771968	7.074213	7.313547	8.155024	8.393233	0.023569	-1.08025	NA	AK013555	Intergenic	NA
CUST_13693_PI419771968	7.487984	7.685805	8.570955	8.784415	0.017614	-1.09079	Vax2os2	NR_002871	Antisense	Vax2
CUST_9036_PI419771968	6.082359	5.883157	7.123452	7.03527	0.029504	-1.0966	NA	AK020501	Sense	Fgf13
CUST_4698_PI419771968	8.149864	8.091274	9.329446	9.119847	0.045264	-1.10408	AK161599	uc008pze	Antisense	Zbtb7b
CUST_2763_PI419771968	7.97777	7.958715	9.030662	9.166678	0.034884	-1.13043	AK020728	uc007pyl	Intergenic	NA
CUST_4137_PI419771968	6.059636	6.435694	7.535807	7.226406	0.046272	-1.13344	AK040197	uc008hrl	Antisense	Mgea5
CUST_6483_PI419771968	8.454265	8.49747	9.521288	9.714966	0.044234	-1.14226	Nrg4	uc009psh	Sense	Nrg4
CUST_6858_PI419771968	4.879615	5.174294	6.319921	6.037432	0.03012	-1.15172	NA	AK003532	Intergenic	NA
CUST_3615_PI419771968	7.456251	7.693717	8.64119	8.860694	0.018694	-1.17596	AK015782	uc008aet	Intergenic	NA
CUST_5023_PI419771968	6.898155	7.047359	8.251934	8.048452	0.014778	-1.17744	AK019643	uc008uuw	Intergenic	NA
CUST_4699_PI419771968	7.032501	6.893016	8.197029	8.106318	0.008702	-1.18891	AK136654	uc008pzf	Antisense	Zbtb7b
CUST_1222_PI419771968	7.442681	7.119411	8.384183	8.567208	0.040455	-1.19465	NA	AK131699	Antisense	Gm20547
CUST_7572_PI419771968	5.863136	5.624995	7.093491	6.791269	0.028204	-1.19831	NA	AK013618	Sense	Gpc5
CUST_690_PI419771968	5.758051	5.664056	6.964487	6.864718	0.003278	-1.20355	NA	AK040157	Sense	Ccdc148
CUST_3827_PI419771968	8.573126	8.833805	10.08684	9.732317	0.038093	-1.20611	AK011222	uc008dte	Antisense	Ppm1b
CUST_384_PI419771968	10.85952	10.979	12.04107	12.22004	0.012443	-1.2113	NA	AK015268	Sense	Pde7a
CUST_2758_PI419771968	11.26339	11.32616	12.50559	12.51225	0.015309	-1.21415	AK020095	uc007pwt	Antisense	Gpld1
CUST_1659_PI419771968	6.284435	6.305886	7.417718	7.61236	0.048187	-1.21988	AK217058	uc007ayq	Sense	1700019D03Rik
CUST_9836_PI419771968	11.43408	11.36469	12.60609	12.66831	0.001504	-1.23781	NA	AK034695	Sense	Cul3
CUST_5631_PI419771968	4.755389	4.742225	5.994827	6.007849	5.47E-05	-1.25253	AK029023	uc009cvm	Antisense	Mgll
CUST_6974_PI419771968	6.935029	6.519393	7.818452	8.163363	0.045575	-1.2637	NA	AK006015	Intergenic	NA
CUST_2613_PI419771968	5.838988	5.889002	7.04854	7.214922	0.028155	-1.26774	AK048798	uc007nws	Antisense	Hif1a
CUST_4244_PI419771968	7.308841	7.483997	8.690666	8.646825	0.033358	-1.27233	2610205E22Rik	uc008jcu	Sense	Mettl11a
CUST_12954_PI419771968	9.083653	9.410965	10.70249	10.34911	0.034124	-1.27849	NA	AK162589	Sense	Slc25a12
CUST_4763_PI419771968	9.247858	9.259761	10.59462	10.4785	0.027239	-1.28275	AK020812	uc008rbq	Sense	A930005H10Rik
CUST_12457_PI419771968	5.523869	5.27281	6.818472	6.544531	0.020733	-1.28316	NA	AK087003	Sense	Itgb6
CUST_4545_PI419771968	6.60162	6.516283	7.745953	7.948979	0.025815	-1.28851	AK133337	uc008ocj	Intergenic	NA
CUST_13398_PI419771968	7.092559	7.163844	8.36925	8.515129	0.012921	-1.31399	NA	U80892	Sense	Nr6a1
CUST_2163_PI419771968	8.88292	9.168165	10.55517	10.12708	0.047765	-1.31558	AK040027	uc007hnf	Intergenic	NA

CUST_11923_PI419771968	7.599043	7.64366	9.029253	8.85636	0.030983	-1.32146	NA	AK080872	Sense	Lpl
CUST_5874_PI419771968	5.080929	5.309145	6.656504	6.399345	0.016888	-1.33289	AB013468	uc009gxh	Sense	Cyth2
CUST_331_PI419771968	12.2499	12.56637	13.97119	13.52386	0.04835	-1.33939	NA	AK013363	Antisense	Slc3a1
CUST_9584_PI419771968	8.232829	8.220497	9.614245	9.542465	0.01403	-1.35169	NA	AK032188	Antisense	Psd2
CUST_12485_PI419771968	4.05713	4.295508	5.71417	5.342735	0.037053	-1.35213	NA	AK087306	Intergenic	NA
CUST_8609_PI419771968	6.085656	5.813448	7.472754	7.134977	0.027479	-1.35431	NA	AK018204	Sense	Kcnd2
CUST_2090_PI419771968	9.498427	9.496484	10.82302	10.89893	0.017644	-1.36352	AK043075	uc007guo	Intergenic	NA
CUST_6859_PI419771968	5.341154	5.0276	6.42057	6.693164	0.023251	-1.37249	NA	AK003662	Intergenic	NA
CUST_7782_PI419771968	8.131332	8.48713	9.821218	9.552543	0.029913	-1.37765	NA	AK015042	Intergenic	NA
CUST_1507_PI419771968	6.794707	7.080521	8.26394	8.376631	0.039312	-1.38267	NA	AK158852	Intergenic	NA
CUST_3629_PI419771968	4.858678	4.454667	5.939932	6.174281	0.043694	-1.40043	Gm1604	uc008aig	Sense	Gm1604b
CUST_11484_PI419771968	6.232878	6.544184	7.894856	7.691397	0.025479	-1.4046	NA	AK052921	Intergenic	NA
CUST_12807_PI419771968	8.985317	8.823867	10.27344	10.36079	0.01139	-1.41253	NA	AK142265	Intergenic	NA
CUST_3507_PI419771968	7.177481	7.263392	8.608763	8.673885	0.002057	-1.42089	AK083150	uc007zfz	Antisense	Zbtb20
CUST_6257_PI419771968	10.77042	10.81974	12.17448	12.33001	0.021096	-1.45717	AK085761	uc009mqg	Antisense	Itfg1
CUST_5489_PI419771968	10.23605	10.33195	11.87096	11.61344	0.033849	-1.4582	BC021953	uc009bfz	Intergenic	NA
CUST_3431_PI419771968	8.525332	8.330298	9.707749	10.08077	0.039817	-1.46644	AK013883	uc007ydb	Sense	1810013L24Rik
CUST_11483_PI419771968	12.96407	13.19336	14.50534	14.59042	0.029055	-1.46916	NA	AK052919	Sense	Trdn
CUST_4115_PI419771968	6.48911	6.696285	8.199094	7.928571	0.016001	-1.47114	AK087295	uc008hlw	Sense	Pik3ap1
CUST_9425_PI419771968	8.874838	9.258336	10.65941	10.41704	0.034421	-1.47164	NA	AK030094	Antisense	Pcdh7
CUST_9051_PI419771968	5.082318	5.38398	6.631175	6.785466	0.029563	-1.47517	NA	AK020529	Sense	Lrba
CUST_2949_PI419771968	8.475695	8.326132	9.716024	10.04135	0.036905	-1.47777	BC096416	uc007sar	Sense	D830030K20Rik
CUST_10802_PI419771968	5.213378	4.807911	6.268041	6.743082	0.042966	-1.49492	NA	AK045293	Sense	Erbb4
CUST_10814_PI419771968	6.84975	6.603746	8.291163	8.178074	0.024339	-1.50787	NA	AK045418	Sense	Phf14
CUST_7669_PI419771968	6.707889	7.072233	8.316463	8.511227	0.035503	-1.52378	NA	AK014217	Antisense	1110001J03Rik
CUST_12043_PI419771968	8.892214	9.16918	10.63765	10.51168	0.028125	-1.54397	NA	AK082229	Sense	Srpk1
CUST_7579_PI419771968	4.705648	4.360875	5.89756	6.291967	0.028146	-1.5615	NA	AK013657	Sense	Kcnip1
CUST_6250_PI419771968	8.39649	8.652998	10.15499	10.04519	0.026424	-1.57535	Dnase2a	uc009mob	Antisense	Mast1
CUST_10891_PI419771968	6.984721	6.659277	8.523796	8.272136	0.019693	-1.57597	NA	AK046572	Intergenic	NA
CUST_2159_PI419771968	7.832741	7.931625	9.559775	9.397037	0.007731	-1.59622	AK005897	uc007hkd	Sense	Tmem194
CUST_1561_PI419771968	12.06245	12.2033	13.84633	13.61288	0.014109	-1.59673	NA	AK165789	Antisense	Chrm2

CUST_11175_PI419771968	6.08238	6.469428	7.792781	7.982059	0.039174	-1.61152	NA	AK049060	Intergenic	NA
CUST_2192_PI419771968	11.14976	11.02139	12.73601	12.68232	0.010345	-1.62359	AK132798	uc007hzc	Antisense	Ramp3
CUST_176_PI419771968	7.904577	8.152061	9.652541	9.719198	0.03512	-1.65755	NA	AK008149	Sense	Acadm
CUST_3642_PI419771968	4.964115	4.494704	6.256717	6.521236	0.04349	-1.65957	AK029100	uc008akr	Intergenic	NA
CUST_9919_PI419771968	8.303838	8.573298	10.078	10.12005	0.046648	-1.66046	NA	AK035420	Intergenic	NA
CUST_9274_PI419771968	8.41072	8.806893	10.42926	10.11138	0.025312	-1.66152	NA	AK021294	Intergenic	NA
CUST_5685_PI419771968	5.310216	5.150031	6.854528	6.97311	0.004927	-1.6837	AK137536	uc009dnn	Sense	Cecr2
CUST_3430_PI419771968	6.035742	6.39141	8.088855	7.706271	0.023529	-1.68399	AK154225	uc007ybf	Sense	4930451G09Rik
CUST_7139_PI419771968	6.648288	6.990353	8.61479	8.43525	0.02786	-1.7057	NA	AK006956	Intergenic	NA
CUST_13146_PI419771968	8.094562	8.315341	10.09758	9.744802	0.023522	-1.71624	NA	BC024137	Intergenic	NA
CUST_226_PI419771968	6.772368	6.82959	8.591175	8.445971	0.012339	-1.71759	NA	AK009611	Antisense	Tmem180
CUST_3643_PI419771968	6.225318	6.104014	7.915885	7.876288	0.012352	-1.73142	AK021075	uc008akv	Antisense	Slc22a3
CUST_5085_PI419771968	6.398611	6.75227	8.485382	8.163811	0.018611	-1.74916	AK035687	uc008vvo	Antisense	Pex14
CUST_247_PI419771968	4.850645	4.687945	6.534897	6.540601	0.029075	-1.76845	NA	AK010374	Sense	Ttl
CUST_13754_PI419771968	5.563372	5.298988	7.31083	7.106334	0.010735	-1.7774	4932435O22Rik	NR_027643	Intergenic	NA
CUST_7327_PI419771968	6.218275	5.676738	7.459818	7.998599	0.043015	-1.7817	NA	AK009503	Antisense	Pla2g6
CUST_11671_PI419771968	8.503444	8.766379	10.61001	10.23356	0.021946	-1.78688	NA	AK076489	Antisense	Itsn2
CUST_6337_PI419771968	6.981634	7.186537	9.082824	8.684256	0.033053	-1.79945	AK085792	uc009nqc	Antisense	Wfdc1
CUST_10618_PI419771968	11.20064	11.40029	13.01161	13.19444	0.00576	-1.80256	NA	AK043387	Sense	Sorbs1
CUST_5185_PI419771968	7.22279	7.091528	9.085994	8.845395	0.014184	-1.80854	AK035982	uc008wvq	Antisense	Kcnk3
CUST_8780_PI419771968	6.374944	6.278246	8.119629	8.186219	0.001951	-1.82633	NA	AK019580	Intergenic	NA
CUST_7329_PI419771968	8.466064	8.305339	10.17443	10.33644	0.003705	-1.86974	NA	AK009508	Sense	Adk
CUST_2586_PI419771968	7.427318	7.686627	9.518565	9.338318	0.010551	-1.87147	AK006973	uc007nny	Intergenic	NA
CUST_5470_PI419771968	6.199523	6.49361	8.143072	8.297301	0.019334	-1.87362	BC062820	uc009axr	Antisense	Gm16039
CUST_2132_PI419771968	5.6195	5.768864	7.628678	7.50887	0.003207	-1.87459	AK145303	uc007hcq	Intergenic	NA
CUST_8410_PI419771968	5.984229	6.178464	8.134996	7.782339	0.023783	-1.87732	NA	AK017413	Intergenic	NA
CUST_811_PI419771968	7.594555	7.873724	9.521899	9.739715	0.010458	-1.89667	NA	AK048740	Intergenic	NA
CUST_3965_PI419771968	4.034646	3.903998	5.77374	5.962822	0.005933	-1.89896	BC028804	uc008fma	Intergenic	NA
CUST_2053_PI419771968	5.822782	6.449909	8.282034	7.795643	0.045898	-1.90249	AK009897	uc007ggl	Antisense	Itgb1bp3
CUST_784_PI419771968	10.48064	10.32126	12.20129	12.42474	0.007532	-1.91206	NA	AK046873	Sense	Cdc37l1
CUST_13315_PI419771968	11.99981	11.81889	13.67718	13.97766	0.015833	-1.91808	NA	BC094579	Intergenic	NA

CUST_9449_PI419771968	8.398626	8.760171	10.26678	10.75635	0.029228	-1.93217	NA	AK030451	Sense	Rbm6
CUST_1322_PI419771968	5.361116	5.442764	7.330243	7.347566	0.009911	-1.93696	NA	AK141772	Antisense	Idh3b
CUST_6242_PI419771968	6.190169	6.382744	8.163077	8.312403	0.00498	-1.95128	AK157878	uc009mlj	Sense	Gm10644
CUST_13207_PI419771968	6.18804	6.43658	8.49913	8.048173	0.032386	-1.96134	NA	BC039959	Intergenic	NA
CUST_4957_PI419771968	4.501895	4.776134	6.726459	6.479204	0.009046	-1.96382	AK002663	uc008ttz	Intergenic	NA
CUST_3565_PI419771968	8.067867	8.313648	10.35566	9.95925	0.023017	-1.9667	AK087725	uc007zsl	Intergenic	NA
CUST_3107_PI419771968	3.977867	3.71987	5.60855	6.059699	0.030551	-1.98526	Mipep	uc007ufh	Sense	Mipep
CUST_10439_PI419771968	8.838531	9.12033	10.89019	11.0502	0.014681	-1.99077	NA	AK041002	Antisense	Tubb6
CUST_3986_PI419771968	5.519095	5.685783	7.607938	7.603979	0.026386	-2.00352	AK172459	uc008frx	Antisense	Pstpip2
CUST_1218_PI419771968	4.756003	4.207123	6.724945	6.286868	0.032099	-2.02434	NA	AK131677	Antisense	Gmnn
CUST_174_PI419771968	7.22125	7.446029	9.453965	9.30634	0.007649	-2.04651	NA	AK008051	Intergenic	NA
CUST_1761_PI419771968	7.958485	8.150694	10.30807	9.89831	0.031605	-2.0486	AK013506	uc007ckw	Antisense	Acmsd
CUST_668_PI419771968	10.47205	10.96308	13.05289	12.501	0.031625	-2.05939	NA	AK038898	Intergenic	NA
CUST_213_PI419771968	8.375289	8.683527	10.82035	10.38214	0.021964	-2.07184	NA	AK009290	Antisense	D17H6S53E
CUST_11222_PI419771968	5.454233	5.430832	7.626699	7.406719	0.031883	-2.07418	NA	AK049981	Sense	Clstn2
CUST_5432_PI419771968	8.075968	8.455598	10.29515	10.42487	0.037582	-2.09423	AK009603	uc009akc	Intergenic	NA
CUST_7282_PI419771968	6.527611	6.5578	8.781361	8.506234	0.039404	-2.10109	NA	AK008862	Intergenic	NA
CUST_8112_PI419771968	6.071375	6.597755	8.624283	8.31398	0.033645	-2.13457	NA	AK016112	Intergenic	NA
CUST_1699_PI419771968	3.89883	4.157677	6.093739	6.236932	0.011998	-2.13708	AK048274	uc007bky	Intergenic	NA
CUST_7318_PI419771968	9.302053	9.856537	12.08373	11.38907	0.043861	-2.15711	NA	AK009349	Intergenic	NA
CUST_4296_PI419771968	9.440764	9.984876	12.06472	11.67802	0.029643	-2.15855	AK007819	uc008jwi	Antisense	Cobll1
CUST_10822_PI419771968	6.803705	6.900685	9.057057	9.069108	0.012584	-2.21089	NA	AK045559	Antisense	2310046O06Rik
CUST_5232_PI419771968	4.519005	4.140282	6.608478	6.481758	0.035569	-2.21547	AK039574	uc008xls	Intergenic	NA
CUST_6461_PI419771968	7.123391	7.355662	9.500545	9.414621	0.017603	-2.21806	Dscaml1	uc009pfx	Sense	Dscaml1
CUST_1018_PI419771968	3.696648	3.339806	5.595165	5.880152	0.012155	-2.21943	NA	AK080710	Sense	BC048943
CUST_9134_PI419771968	6.576732	6.388878	8.553643	8.861928	0.012732	-2.22498	NA	AK020813	Antisense	Dst
CUST_7592_PI419771968	7.028091	6.967996	9.200459	9.393479	0.016115	-2.29893	NA	AK013743	Sense	Mapk10
CUST_483_PI419771968	7.789964	7.900298	10.21305	10.09755	0.001207	-2.31017	NA	AK019197	Sense	Mgst3
CUST_2155_PI419771968	5.054427	5.52933	7.971774	7.34089	0.031757	-2.36445	C78409	uc007hih	Sense	Slc26a10
CUST_7317_PI419771968	9.507999	10.08143	12.02237	12.30863	0.038757	-2.37078	NA	AK009336	Antisense	1600029D21Rik
CUST_4347_PI419771968	6.562963	6.963665	9.404385	8.904472	0.019838	-2.39111	AK132189	uc008kvl	Antisense	Pacsin3

CUST_13250_PI419771968	6.952913	7.528724	9.957798	9.347658	0.029162	-2.41191	NA	BC051480	Intergenic	NA
CUST_8796_PI419771968	7.11807	7.470032	9.49647	9.923341	0.014331	-2.41585	NA	AK019637	Antisense	Mprip
CUST_4996_PI419771968	6.025939	6.620228	9.012012	8.515258	0.026169	-2.44055	AK078255	uc008uhp	Intergenic	NA
CUST_3411_PI419771968	4.357683	3.845377	6.180313	6.912733	0.040371	-2.44499	AK139359	uc007xwq	Intergenic	NA
CUST_11760_PI419771968	5.658741	6.028724	8.384483	8.33491	0.04325	-2.51596	NA	AK078252	Sense	Dlg2
CUST_4662_PI419771968	9.185065	9.033201	11.64199	11.65814	0.017748	-2.54093	AK031097	uc008pmi	Sense	Ppm1l
CUST_778_PI419771968	12.14399	12.2581	14.62789	14.87378	0.011493	-2.54979	NA	AK046581	Sense	Fitm2
CUST_9083_PI419771968	8.254611	8.690213	10.81167	11.23496	0.013913	-2.5509	NA	AK020603	Intergenic	NA
CUST_7036_PI419771968	7.743075	8.024158	10.74711	10.16284	0.036817	-2.57136	NA	AK006445	Intergenic	NA
CUST_4538_PI419771968	6.961963	7.098986	9.576765	9.661385	0.002526	-2.5886	AK052878	uc008obi	Antisense	Sall4
CUST_4084_PI419771968	10.31621	10.63803	13.4271	12.73752	0.046962	-2.60519	AK086006	uc008hbv	Intergenic	NA
CUST_6241_PI419771968	5.230838	5.753952	7.874513	8.336513	0.018114	-2.61312	AK134933	uc009mlf	Intergenic	NA
CUST_219_PI419771968	7.438768	6.913454	10.06582	9.568251	0.0184	-2.64093	NA	AK009418	Intergenic	NA
CUST_13262_PI419771968	6.986783	6.344514	9.049444	9.565885	0.026221	-2.64202	NA	BC054084	Intergenic	NA
CUST_9789_PI419771968	4.135911	4.540052	6.895091	7.0839	0.021679	-2.65152	NA	AK034284	Sense	Creb5
CUST_7264_PI419771968	4.315603	4.545172	6.985136	7.196355	0.003524	-2.66036	NA	AK008598	Antisense	Pcdh1
CUST_7303_PI419771968	5.555565	5.993912	8.758879	8.174359	0.021959	-2.69188	NA	AK009088	Intergenic	NA
CUST_10397_PI419771968	5.497644	5.604847	8.022955	8.510908	0.04739	-2.71569	NA	AK040435	Intergenic	NA
CUST_8579_PI419771968	4.73084	4.598056	7.286512	7.489952	0.00399	-2.72378	NA	AK018012	Sense	Mapk14
CUST_4384_PI419771968	5.90678	6.61284	8.811697	9.188653	0.039751	-2.74037	AK006310	uc008lmt	Antisense	Ccdc34
CUST_4729_PI419771968	7.084018	7.653119	10.39956	9.840275	0.020402	-2.75135	AK052161	uc008qne	Antisense	Polr3gl
CUST_6592_PI419771968	5.345624	6.231148	8.985021	8.108108	0.047445	-2.75818	AK134047	uc009rgt	Antisense	Bfsp2
CUST_4156_PI419771968	4.475409	4.586558	7.351019	7.301858	0.003669	-2.79546	mKIAA1560	uc008hxj	Sense	Gpam
CUST_11126_PI419771968	7.47933	7.808822	10.30632	10.57853	0.006606	-2.79835	NA	AK048684	Intergenic	NA
CUST_9596_PI419771968	5.367318	5.856408	8.656822	8.175284	0.014659	-2.80419	NA	AK032312	Sense	Kcnd2
CUST_2292_PI419771968	7.332396	7.618729	10.35084	10.26464	0.02069	-2.83218	AK149240	uc007jlo	Sense	Pirt
CUST_9798_PI419771968	5.164173	5.355424	8.006865	8.326737	0.009015	-2.907	NA	AK034340	Intergenic	NA
CUST_12257_PI419771968	8.136285	8.600069	11.43608	11.15171	0.015735	-2.92572	NA	AK084755	Sense	Kcnd2
CUST_7918_PI419771968	4.207745	4.364059	6.928682	7.513747	0.048973	-2.93531	NA	AK015455	Intergenic	NA
CUST_7283_PI419771968	4.73696	4.16933	7.647849	7.135782	0.016996	-2.93867	NA	AK008868	Antisense	1700011I03Rik
CUST_6172_PI419771968	8.244566	8.360525	11.31967	11.22533	0.000806	-2.96995	AK040224	uc009lkz	Antisense	Mfhas1

CUST_2494_PI419771968	6.539216	6.885022	9.550309	9.816805	0.00682	-2.97144	AK086245	uc007mmy	Intergenic	NA
CUST_8809_PI419771968	4.973059	4.991781	8.0364	7.992302	0.00103	-3.03193	NA	AK019673	Sense	Magi1
CUST_7328_PI419771968	3.649005	3.389962	6.230311	6.892866	0.042352	-3.04211	NA	AK009506	Antisense	Camk4
CUST_5749_PI419771968	5.423513	5.694732	8.600889	8.675331	0.018968	-3.07899	AK084599	uc009erv	Antisense	Sspn
CUST_9198_PI419771968	5.65015	6.044548	8.577667	9.296727	0.03316	-3.08985	NA	AK021025	Intergenic	NA
CUST_2870_PI419771968	4.173501	4.860521	7.879304	7.356664	0.022589	-3.10097	AK131952	uc007rdj	Intergenic	NA
CUST_7293_PI419771968	6.253864	6.906753	9.809153	9.574258	0.043233	-3.1114	NA	AK008951	Intergenic	NA
CUST_1751_PI419771968	5.168959	4.933283	8.185163	8.163671	0.022905	-3.1233	AK085943	uc007chu	Intergenic	NA
CUST_6690_PI419771968	4.085262	3.921521	6.95175	7.48664	0.036729	-3.2158	mKIAA1575	uc009sqv	Sense	Bcor
CUST_12616_PI419771968	7.816864	8.036139	11.19598	11.13847	0.014526	-3.24073	NA	AK089956	Intergenic	NA
CUST_4022_PI419771968	4.283289	4.652611	7.70846	7.839124	0.020054	-3.30584	BC085508	uc008gcx	Antisense	Drap1
CUST_2296_PI419771968	7.537372	7.842243	10.95791	11.06531	0.015782	-3.3218	Dhrs7c	uc007jnb	Sense	Dhrs7c
CUST_647_PI419771968	5.171337	5.275612	8.561766	8.766587	0.004796	-3.4407	NA	AK036265	Antisense	Bdh1
CUST_7342_PI419771968	5.522897	4.993771	8.596963	8.854873	0.020522	-3.46758	NA	AK009705	Intergenic	NA
CUST_2098_PI419771968	5.442866	5.986299	9.06716	9.305318	0.02437	-3.47166	AK087886	uc007gwe	Antisense	Cradd
CUST_5743_PI419771968	7.195631	8.101384	11.51341	10.75607	0.029535	-3.48623	AK039595	uc009erd	Antisense	Casc1
CUST_4456_PI419771968	4.010314	4.466153	8.206489	7.545438	0.017155	-3.63773	AK085768	uc008mqp	Antisense	Banf2
CUST_53_PI419771968	11.12551	11.34905	15.00911	15.59551	0.025592	-4.06503	NA	AK003803	Sense	Dgat2
CUST_1089_PI419771968	7.556989	7.858784	11.7501	12.0308	0.002479	-4.18256	NA	AK084656	Intergenic	NA
CUST_6072_PI419771968	10.00078	10.47062	14.51285	14.83331	0.00675	-4.43738	AK041267	uc009kdm	Antisense	Dhx32

Table S6 Sequences of the full-length lncRNA-*Safe*.

Red sequence represents newly amplified sequence by 5’ RACE assay, blue sequence represents newly amplified sequence by 3’ RACE assay.
**AGAGCCCAGCAAGTAACCCTTTAAGGTGACATCTAGTGACTCAGAGCACTCTTAGATTG<br>ATTACCTCAAGGCGGAAGGAGAAGGTGGAAGTACAGTGCCTCGCCTCTCTTTTACCCTG<br>AGCCAGTGGGAACTGGTGGGAACAGATAGGGAACAGCCTGGGAGAGAGGATGAAGAG<br>AAGGAAGAAAGCCCGGAGAGAAGGAGGAACGAGAAAGGGGCCATTTCCCAGAAGGTC<br>CGGAGACCCTGCCTCCGTCCGGGGCCGGCTTACATCCCTGTGGGCGGAGTCTTGGAGA<br>ACAGACCGCGTCTCGCGCTTGCGGGGCC**ACATCCTCAGGACTCGTGCGGGGTCCCTGGCT<br>CTCACCCCGCGGGTACCTGCTCGAGCTGCGGGGCTACCTCCAGCACGGCCGCCGCTTTGAGC<br>TCCGCCATGATCGTCGCCGCGTCGGATCCCCGCCGGGAGAGTGTAGCAGAGTCCGCGTTGGA<br>GCCGAGCTGACAGTGGCCGGGCGGGTGGCCTGGCTGTGGCTGCAGTGGTACCTCCTCCGGAG<br>CCCAGGTCACCATGCACTGCGCACCTCCCTAGCGTAGTGAGGGACTCCTCAGTGTGGCATGG<br>GCACGTTCTGCGGGTCCTCCTACATGATGAAAGGTCCTGCGTCCCACCACGCCCTGTACTCTC<br>TGCTGGAACCAGAGGTGACTGGGCTGGAGGAGGGTCCGGAACAGAACTTAAGTATGGAAGG<br>ACTTACCACATTCACAATGGCTGCTCTTCTCTAAGAATCTCCAGCAATACACAGCAGGACTGT<br>GAGGCACAGTTCAGAGTCCCATTGATATGTAACTGAATTGCTGAGATGGACCAATGAGAGCCT<br>TCGAAGTCTCTGCTGTCTGCAGGAGCAGTGGCCACGAATCTGGCTGAAATGTGGGGTACATG<br>CTAATGTATGGGGAGACCAGTCTTAAACCACTGGAGTAGCTGGTAACTTCAACCTGCTGTGGG<br>GAGGACTGGGCCTCCTGAGAAGGAAATGACAGAGCTCCCTCCAAGGTCGTTGGTCAAAGGA<br>GACATGACAGAAGACAAAGAATAGGTACAACAAGATGTAAAGTTTATATACAAAGAATATAAT<br>GTTTATCTGAAATATTTACAGTGTCGGTTAAAGCAATATTTTTACAACTTTTCCAGGTCAACTAC<br>TACGTATATTAC**AGGTAAGCTACAATGATTTAGTTTGCAAAAAAAATGATTAAAAAATTGAA<br>TTAAAAGATGTTTTAAACAAAGCTTAAAATACTCGTCAATTTATAGACTGTATTCACTTTTT<br>TATTTG**AGGGCATCATGCAATGAGGAATGGTTACTGTGTGCTTATAAACTGAAGTTCTGTAGC<br>TGGGATGGGAACGCATGCAATGGTCAGAGCTCATGAGGGCATAAAACGAGCAACGAAATGTT<br>TGAAGACTTAGATGCCGATTCTCAGGCTGGAACTGAGGAAGACTTTGAGATAACAGCCAGCT<br>TTATCTCAACTAAGTCTGGGACCCACAAGCTTAGACCACAGAGGG

Table S7 List of predicted open reading frame in *Safe* sequence.

Label	Strand	Frame	Start	Stop	RNA length (nt)	Protein length (aa)
ORF-1	+	3	168	410	243	80
ORF-2	+	1	595	756	162	53
ORF-3	+	2	1406	>1516	111	36
ORF-4	+	3	1353	1421	69	22
ORF-5	+	2	419	484	66	21
ORF-6	+	3	999	1058	60	19
ORF-7	+	2	839	895	57	18
ORF-8	+	3	1422	1463	42	13
ORF-9	+	2	908	949	42	13
ORF-10	+	3	918	956	39	12
ORF-11	+	3	1305	1337	33	10
ORF-12	-	2	322	41	282	93
ORF-13	-	3	549	352	198	65
ORF-14	-	3	219	61	159	52
ORF-15	-	3	909	796	114	37
ORF-16	-	1	1382	1278	105	34
ORF-17	-	2	1039	959	81	26
